# Tools for the identification of variable and potentially variable tandem repeats

**DOI:** 10.1186/1471-2164-7-290

**Published:** 2006-11-15

**Authors:** Colm T O'Dushlaine, Denis C Shields

**Affiliations:** 1 Bioinformatics Core, Molecular and Cellular Therapeutics, Royal College of Surgeons in Ireland, Ireland; 2 UCD Conway Institute, University College Dublin, Dublin 4, Ireland

## Abstract

**Background:**

Tandem repeat arrays showing variation between sequences within a population, between strains or across species may have functional effects. The increasing availability of genomic sequence data makes routine description of observed variation possible, creating a need for tools to describe such variability.

**Results:**

We present a set of programs that facilitate the identification of tandem repeats showing variation across multiple sequences or genomes, and the prediction of potentially polymorphic tandem repeats. The VNTRfinder (Variable Number of Tandem Repeats finder) program enables the detection of sequence length variation between arrays of inter-specific or intra-specific tandem repeats. In the absence of comparable sequences to explore observed variation, predictions are provided describing which tandem repeats are more likely to be variable, to help guide and focus further experimental evaluation.

**Conclusion:**

These tools represent a resource for researchers interested in tandem repeats in nucleotide sequences that are most likely to be of clinical and evolutionary interest. The tools are available at . Downloadable versions for UNIX/LINUX and WINDOWS which permit the consideration of longer and more numerous sequences are also available.

## Background

The identification of tandem repeats exhibiting or with the potential to exhibit length variation is of considerable importance to medical and evolutionary-based research. Repeat variability may be associated with important phenotypes. Lack of changes in repeats between evolutionarily distant species may reflect high functional constraint, suggesting the functional importance of repeats within a given genomic region. Denoeud and Vergnaud [1] have described a web-based resource [2], which displays pre-computed tandem repeat length variations among bacterial strains. Another resource is TRDB, Tandem Repeats Database [3]. This versatile resource allows users to upload sequences, detect tandem repeats using the Tandem Repeats Finder algorithm and perform actions such as extracting flanking sequences and predicting primer sequences. In particular, TRDB provides a graphic viewing of internal variations along the tandem array in a coordinated way for multiple alleles. Here, we present software that allows users to upload and detect tandem repeats, but that is primarily concerned with the prediction and detection of tandem repeat copy-number variation and resulting length polymorphism.

Our objectives were to provide software for researchers interested in studying Variable Numbers of Tandem Repeats (VNTRs). We set out to provide a platform for comparing available sequences to rapidly identify repeat copy-number variants within tandem repeats (VNTRfinder software), and to provide predictors of polymorphism (based on rules generated by previous work [4,5]: PolyPredictR software).

Both VNTRfinder and PolyPredictR are written in PERL and designed to run in UNIX. The Tandem Repeats Finder (TRF) [6] and e-PCR [7] programs are called by these scripts and their results are parsed and included in the final output. TRF is commonly used to detect repeat in nucleotide sequences. It identifies likely tandem repeats by looking for clusters of small matching words (k-tuples) separated by a common distance. Dynamic programming alignment is used to confirm a repeat and yields a consensus pattern and tandem array. Parameters allow modification of the alignment weights, minimum alignment score to report, and maximum pattern size detected. e-PCR is a program commonly used to recover sequence-tagged sites (unique genomic landmarks) by searching for sub-sequences that closely match the PCR primers for these sites. If these sub-sequences have the correct order, orientation and spacing and could thus presumably lead to the amplification of a PCR product of the correct molecular weight, the match is reported. The scripts that call TRF and e-PCR are implemented online through a CGI interface and are also available for download. Web graphics are generated in HTML where the width of the images is proportional to the length of the tandem repeat with respect to the overall length of the sequence.

## Implementation

### Program: VNTRfinder

VNTRfinder uses the TRF program [6] to detect repeats. Both variant and invariant repeats are reported, the former defined as a tandem repeat from a reference sequence that is found in a target sequence by VNTRfinder and observed to be of different length. The e-PCR [7] program is used to match known repeat regions in a query sequence to the apparently matching region in the target. The program permits variation in the length of the chosen flanks, and the number of permitted mismatches. The e-PCR wordsize parameter is set to 0 so that a maximum number of candidate matches are considered. The user can specify TRF repeat detection parameters and paste or upload two files with sequences of interest. The first file contains the reference sequence(s), the second the target sequence(s) – the sequences across which to look for repeat variation, of which an unlimited number can be entered. TRF is run on the reference(s) and any redundancy from overlapping repeats is eliminated according to the method described by Denoeud and colleagues [8]. For VNTRfinder, flanking regions of each tandem repeat identified in the reference sequence(s) are then aligned to the related regions of target sequence(s). For cases where there is one reference and one target sequence, there is the option to automatically run the search in the reverse direction.

For computational efficiency, the method implemented requires that there are no insertions or deletions in the TR flanks. This has the drawback that for more distantly related homologous repeats, the program will miss them. This feature of our approach will be disadvantageous in certain contexts, i.e. when the flanks of tandem repeats are strongly diverged, and contain indels. However, for many practical applications this is not a major issue: where it is a problem, the alternative BLAST based approach of Denoeud and Vergnaud is more optimal.

A mismatch parameter defines the number of permitted mismatches in flanking sequences. In locating the best matching sequence, a search is conducted by starting with zero mismatches, which is increased, if necessary, until a single, unambiguous match between reference and target sequences is obtained, or until the value for the maximum mismatches permitted is reached. If two regions of an identical degree of base similarity are detected, then the results are excluded. The motivation for taking this approach, rather than systematically extending the repeat flanks until an apparently unique orthologous region is defined, was as follows; a search of 215,000 repeats from the human genome against itself with a flanklength of 20 revealed approximately 2% that had more than one match. A simple expectation would be ~1 × 10^-7^. Since the great excess of such matches are likely to represent recently duplicated regions of the genome, simply extending the flanks to find the better of the two matches may be prone to erroneous matching of paralogous regions. By avoiding such extension the user is less likely to be misled by such mismatches. In our experience with the smaller bacterial datasets, systematic extension of flanks to identify a match had little effect on the ability to match many additional TRs.

The speed of VNTRfinder decreases with the number of sequences being searched against; as each repeat in the references(s) is searched against each target sequence, the more targets, the longer the search will take because each reference repeat must be searched against each target. For instance, a search of tandem repeats across the genome of *Mycobacterium tuberculosis *H37Rv against the genome of *Mycobacterium tuberculosis *CDC1551 took approximately 32 minutes for a dataset of 690 repeats detected using Tandem Repeats Finder default parameters. When *Mycobacterium bovis *was added as an additional target sequence, the search time increased to 62 minutes and when *Mycobacterium leprae *was added as another target, the search time increased to 127 minutes computational time. Also, increases are proportional to the degree of evolutionary divergence of the tandem repeat flanks

It is possible to scan the identified related region of the reference sequence between the flanks for repeats using TRF to enrich for length variations that represent a genuine tandem repeat array length difference rather than alternative splices, genome rearrangements etc. This is done by specifying to keep results where the hit "*represents length difference consistent with change in the repeat copy-number*" is selected. In this instance, the repeat unit length and motif will be the same in both reference and target (if this option is not selected, these may differ). Specifically, the sequence of the hit is scanned with TRF and tandem repeats are checked to ensure that the observed copy-number agrees with the expected one, given the length of the hit tandem array and the length of the repeat unit as follows:

For cases where the length of the hit tandem array is greater than that of the reference, we calculate *I*, a measure of the inconsistency of the variant with any multiple of the repeat copy number. This is calculated as

I=ExpV−ObsVExpV−ObsR
 MathType@MTEF@5@5@+=feaafiart1ev1aaatCvAUfKttLearuWrP9MDH5MBPbIqV92AaeXatLxBI9gBaebbnrfifHhDYfgasaacH8akY=wiFfYdH8Gipec8Eeeu0xXdbba9frFj0=OqFfea0dXdd9vqai=hGuQ8kuc9pgc9s8qqaq=dirpe0xb9q8qiLsFr0=vr0=vr0dc8meaabaqaciaacaGaaeqabaqabeGadaaakeaacqWGjbqscqGH9aqpdaWcaaqaaiabdweafjabdIha4jabdchaWjabdAfawjabgkHiTiabd+eapjabdkgaIjabdohaZjabdAfawbqaaiabdweafjabdIha4jabdchaWjabdAfawjabgkHiTiabd+eapjabdkgaIjabdohaZjabdkfasbaaaaa@4533@

when the length of the hit is shorter than that of the reference, this is represented as

I=ExpV−ObsVExpV
 MathType@MTEF@5@5@+=feaafiart1ev1aaatCvAUfKttLearuWrP9MDH5MBPbIqV92AaeXatLxBI9gBaebbnrfifHhDYfgasaacH8akY=wiFfYdH8Gipec8Eeeu0xXdbba9frFj0=OqFfea0dXdd9vqai=hGuQ8kuc9pgc9s8qqaq=dirpe0xb9q8qiLsFr0=vr0=vr0dc8meaabaqaciaacaGaaeqabaqabeGadaaakeaacqWGjbqscqGH9aqpdaWcaaqaaiabdweafjabdIha4jabdchaWjabdAfawjabgkHiTiabd+eapjabdkgaIjabdohaZjabdAfawbqaaiabdweafjabdIha4jabdchaWjabdAfawbaaaaa@3F36@

where *ExpV *is the expected copy-number for the variant (given the length of the variant and the length of the repeat unit), *ObsV *is the observed copy-number for the variant, as estimated by TRF, and *ObsR *is the observed copy-number for the reference repeat. This gives a distribution close to 0 for variations consistent with a change in repeat copy-number and close to 1 for other variants. Only variants with a value of *I *below 0.5 are retained. The equations serve to describe how closely a length variant matches the expected lengths seen from changes that represent precise changes in copy-number. The cut-off chosen is arbitrary, however, the vast majority of datapoints lie quite close to 0 or 1, and therefore alternative choices of the chosen cut-off are unlikely to alter the results obtained. It is important to note that this option represents a stringent search for repeat variation. The option to search for other types of variations is thus also provided, which identifies significantly more matches.

Descriptive statistics are gathered to describe the reference repeat and the extent to which the repeat varies across the reference and target sequences. These are the identifier of the reference sequence containing the repeat(s), the repeat unit length and tandem array length, the repeat unit sequence and tandem array sequence, the start and stop coordinates of the repeat in the reference sequence, the repeat copy-number, the left and right flanking sequence of the repeat, the total tandem array length of the reference repeat and all the hits, represented as a "population" of tandem array lengths, e.g. 26|28|26, and the number of mismatches tolerated in aligning the flanks to the target sequence.

Summary statistics that describe variability detected are also provided. These include a simple binary metric describing whether or not a repeat was observed to be variable and also a heterozygosity score (equivalent to "gene diversity" [7]) that describes the variability of the repeat in the population of sequences analyzed. The standard deviation of the heterozygosity is also given, which is clearly excessively large in the case of a variant detected between only two sequences, but becomes a useful statistic to establish the reliability of the heterozygosity in the situation where the user has as input a large number of sequences, which have a large number of allelic variants. The standard deviation and standard error of all observed tandem repeat array length alleles is also provided, which provides an indication of the spread of allele sizes. In many cases, the heterozygosity is not particularly informative unless derived from a large sample size and therefore close attention should be paid to the standard error of the repeat array length alleles. The lengths of all unique tandem array lengths and their frequency of occurrence are also provided. Examples of the output files can be viewed on our website.

### Program: PolyPredictR

PolyPredictR predicts potentially polymorphic tandem repeats using simple rules previously described [4,5]. The first set of rules, described by Wren and colleagues [4] use information on the length of the tandem repeat, its homogeneity and the copy-number of the repeat unit to predict polymorphism. Specifically, if a repeat is 100% homogenous (all units are identical), the copy-numbers 12, 6.5, 5.5, 4.5, 3.5 and 2.5 represent thresholds beyond which the repeats of unit length 1, 2, 3, 4, 5–9 and >=10 are predicted to be potentially polymorphic, respectively. If all units are not identical, these rules may not be applied. An earlier implementation of these rules does tolerate imperfect repeats (>90% homogenous) [9] but has higher false positive rates. These rules are also implemented by PolyPredictR. The second set of rules, described by Naslund and colleagues [5] uses a larger number of criteria as a predictive model. These include copy-number, entropy (summarises the percentage of different nucleotides in the repeat), GC dinucleotide bias and percentage match between repeats in the tandem array. These very simple rules provide a crude indicator, but the authors' validations of their efficiency have been relatively limited. Therefore, the utility of these predictors are not well understood and should be treated with some caution. Other attempts to understand the relationship between polymorphism and repeat sequence characteristics have had limited success, highlighting the difficulty of obtaining a set of rules applicable and relevant to all genomes [1,8,10]. Notably, it has been shown that sensitivity of various measures, such as the total length of the tandem repeat, the percentage matches between adjacent copies of the repeat unit, and the GC content of a tandem repeat, while significant predictors of polymorphism, can vary in their predictive power between different species [1,10].

## Results

### VNTRfinder: comparison to Denoeud and Vergnaud method

We compared VNTRfinder results to those of Denoeud and Vergnaud [1] for a comparative survey of two strains of the prokaryotes *Mycobacterium tuberculosis *(CDC1551 vs. H37Rv) and *Neisseria meningitidis *(MC58 vs. Z2491). The settings for VNTRfinder were as follows: TRF minscore, match, mismatch, indel and maxperiod scores set at 20, 2, 3, 5 and 500 respectively. A number of different flanklength, mismatch and hit retention parameters were evaluated and the results were compared to both the results of Denoeud and Vergnaud and to the original dataset of repeats detected (Table 1, Table 2).

The first observation (Table 1) is that for the repeats that were successfully matched by both methods in *Mycobacterium tuberculosis*, they agreed quite strongly in their definition of whether or not they were variant (95% of Denoeud variants were also classed as variants by VNTRfinder; Table 2 (the percentage is 62% (72/116) for VNTRfinder variants also classified as variants by the Denoeud method.

For the more distantly related *Neisseria meningitidis *comparison, VNTRfinder again matches more repeats (69% versus 51% matched by the Denoeud method) (Table 1). In comparison to the *Mycobacterium tuberculosis *search, fewer VNTRs identified by Denoeud and Vergnaud are also detected by VNTRfinder (87% in *Mycobacterium tuberculosis *compared to 82% in *Neisseria meningitidis*) (Table 2). This is most evident when the check to retain only repeat variants that are consistent with a change in copy-number is applied (82% reduces to 38%). There is also an increase in the percentage of repeats reported as variant by Denoeud but as invariant by VNTRfinder.

Why does VNTRfinder miss some of the repeats reported by Denoeud and Vergnaud? This appears to be mainly due to the presence of gaps in the flanks. VNTRfinder does not tolerate gaps when matching flanking sequences to a target sequence. By matching flanking sequence (combined upstream and downstream flanks summing to 200 bases) between the *Neisseria meningitidis *species based on the coordinates reported by Denoeud and Vergnaud, we found that 42% (1487/3527) of repeats that were successfully matched between species using VNTRfinder had gaps whereas 56% (260/464) of repeats not successfully matched had. If we consider only repeats with no gaps in the flanking sequences when matched between species, then our method detects 91% (2040/2244) of repeats detected by Denoeud and Vergnaud (using the parameters of flank 40, mismatch 10, reporting of all variations). In terms of variants shared between datasets, when there are no gaps in the flanks, we identify 75% of their variants whereas we only identify 27% when there are gaps. Thus, VNTRfinder is better suited to matching homologous repeats with reasonably stable flanking sequences. The example comparison between the highly diverged strains of *Neisseria meningitidis *is likely to be close to or well beyond, the limits of evolutionary distance that is of interest to most researchers; we included this extreme example to clearly highlight the operational differences between different search strategies.

A number of repeats in *Neisseria meningitidis *(18 out of 3533) were detected by both methods, but were reported as variant by one method but not by the other. Dot-plots were made of the sequences involved in these 18 incongruent classifications [see Additional file 1]. Of the two variants detected by VNTRfinder that Denoeud failed to detect, in one case there was a conflict in the choice of homologous segments, in the other the variant was an indel in the array flank/repeat boundary. Of the 16 that Denoeud detected and VNTRfinder did not, 1 represented a conflict in choice of homologous segments, 12 involved length variants that did not lie within the repeat array (often very poorly aligned regions) and 3 involved indels at the flank/repeat array boundary. Thus, most of these conflicting results highlight regions that are difficult to compare.

We compared the results of VNTRfinder to 31 previously reported markers that were variant between *Mycobacterium tuberculosis *strains H37Rv and CDC1551 (summarised in [11]). For all but one of the markers, VNTRfinder also reported them as variant and the reported length difference was identical. The marker missed, Mtub24, is among the 3 markers that were noted as being problematic [11], i.e. repeat variant where the repeat unit is difficult to define. The Denoeud et al. [1] web resource [2] reported all 31 as variant. The same 8 of the 9 markers invariant between *Mycobacterium tuberculosis *H37Rv and CDC1551 according to Le Fleche et al. [11] were reported as invariant TRs by both methods. One – MIRU24 – was not reported as a detectable TR by either method. This is not surprising since only one repeat unit is present in the two *Mycobacterium tuberculosis strains *[11]. In general, repeats reported by either the Denoeud or VNTRfinder methods were shorter than those reported by the Le Fleche study because of the parameters used to detect tandem repeats with the TRF algorithm.

### VNTRfinder: general considerations

As with any alignment method, it is important to stress that parameter choice for flanks and mismatch tolerance should reflect the relationships between the sequences being analysed. For instance, if the user is analyzing sequences from two different genomes where the divergence rate is high, some repeats may be missed. Therefore, if the user is interested in a specific repeat and it has not been reported, possibly as a result of the emergence of indels in the flanking sequences, we recommend decreasing the flank length and/or also increasing the mismatch tolerance. These parameter choices will increase the time needed for the search to complete but will increase the likelihood of obtaining a hit, particularly in the case of the flank length choice, as shorter flanks are less likely to overlap sequences containing gaps between the sequences being aligned. For instance, if comparing mammalian sequences of around 80% identity, flanklength and mismatch parameter settings of 10 and 2 might represent more appropriate defaults for searching.

### Program: PolyPredictR

There are a number of caveats to the use of these rules. Firstly, the rules were inferred from human variants and thus may not necessarily hold for other species; secondly, many observed variants do not qualify with these rules because even very short repeats (repeats not covered by these rules, where tandem array length is less than 12 nt) have been observed to be variant [12]; thirdly, many predicted variants may not be variant, with approximately a third of the predictions not being commonly variant using the Wren et al. rules [4], and fourthly, rules present by Naslund and co-workers [5] only pertain to the prediction of potentially polymorphic repeats of units of six or more bases in length. Nevertheless, the rules serve as an initial guide and have some predictive power. They are therefore integrated with VNTRfinder on our web server in addition to being provided as a separate standalone application. For example, all 12 loci reported by [11] that were not polymorphic between *Mycobacterium tuberculosis *H37Rv and CDC1551 were also reported as invariant by VNTRfinder. We investigated which repeats would be predicted to be variant between these two strains; of 4362 repeats with unit length longer than 9 bp, 10 were predicted by the Wren et al. rules as being polymorphic. (In total, 63 repeats of unit length > 9 were reported as variant by VNTRfinder.) Only 0.41% (18) were predicted as polymorphic by either these rules or the POMPOUS earlier implementation of these rules [9]). Among the 30 known repeats reported as variant between *Mycobacterium tuberculosis *H37Rv and CDC1551 [11] and detected by VNTRfinder, 7 were predicted as variant by the Wren rules and an additional 7 were predicted as variant using the POMPOUS rules. Thus, in total, 14 of the 30 repeats (47%) were predicted by either method to be polymorphic. Thus, in spite of the fact that the Wren and POMPOUS rules were designed for mammalian genomes, they may have some more general utility, since they seem to favour repeats chosen by *Mycobacterium tuberculosis *researchers. The two advantages of the Naslund approach are firstly that it provides the probability that the TR is variable, rather than a binary score; secondly, it is trained specifically on minisatellite repeats, so that it may well provide a better prediction than the Wren et al rules, though obviously it is restricted to TRs with a unit length of 6 or greater.

In light of the above, we recommend VNTRfinder and the identification of variation by matching repeats between homologous sequences as a more robust approach to detecting potential repeat polymorphisms, but in the absence of such sequences, PolyPredictR is an available useful tool for indicating the potential polymorphism of a repeat.

A visual overview of results highlighting repeats, variant repeats and potentially polymorphic repeats is also provided from which users can link to the relevant results files.

## Conclusion

VNTRfinder compares well to an existing resource of repeats matched between bacterial species [1]. The software is available to run on data for which no pre-computed results are available on the web, and uses software that is more adept at aligning low-complexity regions [7]. The particular method applied is complementary to that used in generating existing pre-computed datasets, missing some variants detected by the Denoeud method, but detecting other variants. In practice, a completely exhaustive search for repeat variants is rarely practical, since in the twilight zone of similarity it is difficult to distinguish efficiently between truly homologous and independent repeats. In essence, the BLAST approach relies on longer range similarity around the repeats, and our method relies on very local similarity. In sequences prone to substantial short range re-arrangement, such as promoter regions, and in low complexity regions our method may prove more efficient, while in more stably evolving sequences the BLAST approach is likely to be more sensitive. VNTRfinder allows filtering to only report repeat variants representing length changes consistent with changes in the copy-number of the tandem repeat unit detected in the reference, as in [2], with the additional check that the bases differing in the insertion/deletion event actually correspond to the tandem repeat unit. The method can also consider multiple sequences and estimate heterozygosity for a tandem repeat locus. Together, these resources will assist in the identification of potentially polymorphic tandem repeats or repeats variable among homologs, paving the way for experimental confirmation and functional analyses of the implications of this variability.

## Availability and requirements

**Project name: **VNTRfinder and PolyPredictR

**Project home page: **

**Operating system(s): **(1) Web interface, (2) UNIX/LINUX (downloadable version), (3) WINDOWS (downloadable version)

**Programming language: **PERL

**Other requirements: **Downloadable versions require PERL

**Licence: **GNU GPL

## Authors' contributions

CTO developed and tested the programs. DCS assisted with program design and the writing of the manuscript. Both authors read and approved the final manuscript.

## Supplementary Material

Additional File 1 Incongruent results for VNTRfinder versus the method described by Denoeud et al. [1]. Dot-plots for 18 instances of differences in reported variability between the two methods in *Neisseria meningitidis *from a total of 3533 repeats matched between methods. Click here for file

## Figures and Tables

**Table 1:** Repeats detected in strains of *Mycobacterium tuberculosis *and *Neisseria meningitidis*.

	Method*	Total**	**Number of variants detected**
**Total repeats detect by TRF in *Mycobacterium tuberculosis *CDC1551**		19411	
	**Denoeud**	12542	130
	
**Repeats matched to *Mycobacterium tuberculosis *H37Rv**	**VNTRfinder (40, 10, all variants)**	15964	116
	**VNTRfinder (40, 10, multiple of unit)**	14490	61

**Total repeats detect by TRF in *Neisseria meningitidis *MC58**		8419	

	**Denoeud**	4299	173
	**VNTRfinder (40, 10, all variants)**	5807	177
	
**Repeats matched to *Neisseria meningitidis *Z2491**	**VNTRfinder (40, 10, multiple of unit)**	2504	22
	**VNTRfinder (20, 4, all variants)**	5822	174
	**VNTRfinder (20, 4, multiple of unit)**	2516	21
	**VNTRfinder (10, 2, all variants)**	5382	301
	**VNTRfinder (10, 2, multiple of unit)**	2338	9

* VNTRfinder settings are given respectively in parentheses as the flanklength allowed, mismatch allowed, and whether or not repeat length differences from the query were required to be multiples of the tandem repeat unit.

** Totals shown are those that align perfectly with the flanks of a repeat originally reported by TRF.

Proportions of total repeats originally detected in *Neisseria meningitidis *MC58 and *Mycobacterium tuberculosis *CDC1551 that were successfully matched by the different methods to *Neisseria meningitidis *Z2491 and *Mycobacterium tuberculosis *H37Rv respectively. 'all variants' refers to the VNTRfinder option to report any detected variation and 'multiple of unit' refers to the option to only report variations where the length variation is consistent with a change in the repeat copy-number. The number of variants detected is also highlighted.

**Table 2:** Comparisons between VNTRfinder and the method described by Denoeud and Vergnaud.

***Mycobacterium tuberculosis *(99.3% identical)**					***Neisseria meningitidis *(48.74% identical)**				
	**Total**	**% Denoeud aligned TRs aligned by VNTRfinder**	**% of Denoeud VNTRs called by VNTRfinder as variant or non-variant**	**% of Denoeud non-variants called by VNTRfinder as variant or non-variant**		**Total**	**% Denoeud aligned TRs aligned by VNTRfinder**	**% of Denoeud VNTRs called by VNTRfinder as variant or non-variant**	**% of Denoeud non-variants called by VNTRfinder as variant or non-variant**

**Denoeud v. 40, 10, all variants**					**Denoeud v. 40, 10, all variants**				

total repeats matched	10860	86.59			total repeats matched	3533	82.18		
DEN^+^, VNTRfinder^+ ^*	72		94.7		DEN^+^, VNTRfinder^+^	69		81.2	
DEN^+^, VNTRfinder^-^	4		5.3		DEN^+^, VNTRfinder^-^	16		18.8	
DEN^-^, VNTRfinder^+^	0			0	DEN^-^, VNTRfinder^+^	2			0
DEN^-^, VNTRfinder^-^	10784			100	DEN^-^, VNTRfinder^-^	3446			100
									

**Denoeud v. 40, 10, multiple of unit**					**Denoeud v. 40, 10, multiple of unit**				

total repeats matched	10160	81.01			total repeats matched	1615	37.57		
DEN^+^, VNTRfinder^+^	39		92.9		DEN^+^, VNTRfinder^+^	9		75.0	
DEN^+^, VNTRfinder^-^	3		7.1		DEN^+^, VNTRfinder^-^	3		25.0	
DEN^-^, VNTRfinder^+^	0			0	DEN^-^, VNTRfinder^+^	0			0
DEN^-^, VNTRfinder^-^	10118			100	DEN^-^, VNTRfinder^-^	1603			100

* '+/-' indicate repeats reported as variants/non-variants.

Repeats were matched between the results reported by VNTRfinder and those reported by the method of Denoeud and Vergnaud.
