# Supplementary material for: Tools for the identification of variable and potentially variable tandem repeats
Source: BMC Genomics. 2006 Nov 15;7:290. doi: 10.1186/1471-2164-7-290 (PMC1654160; doi:10.1186/1471-2164-7-290)
Supplement: Additional File 1 — Incongruent results for VNTRfinder versus the method described by Denoeud et al. [1]. Dot-plots for 18 instances of differences in reported variability between the two methods in Neisseria meningitidis from a total of 3533 repeats matched between methods. [file 1471-2164-7-290-S1.pdf]

			Denoeud results	VNTRfinder results	Differences reported		Comments/ reasons for conflict
Repeat number	***Repeat Start MC58***	***Repeat stop MC58***	***Hit start-stop MCZ2491***	***Hit start-stop MCZ2491***	***Denoeud reported difference***	***VNTRfinder reported difference***	
1	23260	23284	256713-256734	253305-253409	3	0	
2	24341	24365	257316-257493	257316-257340	153	0	
3	281145	281183	2163317-2163596	2163558-2163596	241	0	
4	370747	370757	2074671-2074675	2074671-2074681	6	0	
5	370778	370800	2074628-2074644	2074628-2074650	6	0	(c.f. repeats 7,10)
6	598226	598240	745362-745376	745363-745376	0	1	
7	613994	614016	2074628-2074644	2074628-2074650	6	0	(c.f. repeats 5,10)
8	794146	794168	941942-941963	941942-941964	1	0	
9	1340997	1341015	1432907-1432940	1432907-1432925	15	0	
10	1443509	1443531	2074628-207644	2074628-2074650	6	0	(c.f. repeats 5,7)
11	1452043	1452078	1535314-1535353	1535314-1535349	4	0	
12	1487085	1487267	1573652-1573834	1573215-1573400	0	3	
13	1527328	1527375	1612372-1612414	1612372-1612419	5	0	
14	1604964	1605008	1741676-1741702	1741676-1741720	18	0	(c.f. repeat 15)
15	1694228	1694275	1741676-1741705	1741676-1741723	18	0	(c.f. repeat 14)
16	1822610	1822663	1934569-1934756	1934569-1934622	134	0	
17	1975357	1975387	558555-558771	558555-558585	186	0	
18	1985578	1985598	547879-547871	547859-547879	12	0	

Reported indel does not appear to lie in tandem repeat array
Very poorly aligned region
Problematic region – arises more than once as a hit
Indel at start of flanking region/end of repeat array
Both methods hit different and/or difficult to align regions

1. **For each repeat, the first plot represents a plot of the repeat and 300nt left and right flanks for the repeat from MC58 (x-axis) versus the hit plus 300nt flanks from Z2491 (y-axis)**

2. **Repeat regions are highlighted by a box**

**Repeat start-stop: 23260-23284**<br>**Unit: GTC**<br>**Tandem array: GTTGTCGGCGGCGACGGTGTCGTCG**<br>**Denoeud report variant of 3**<br>**VNTRfinder report variant of 0**

Denoeud: 23260-23284 (MC58) versus 256713-256734 (Z2491)

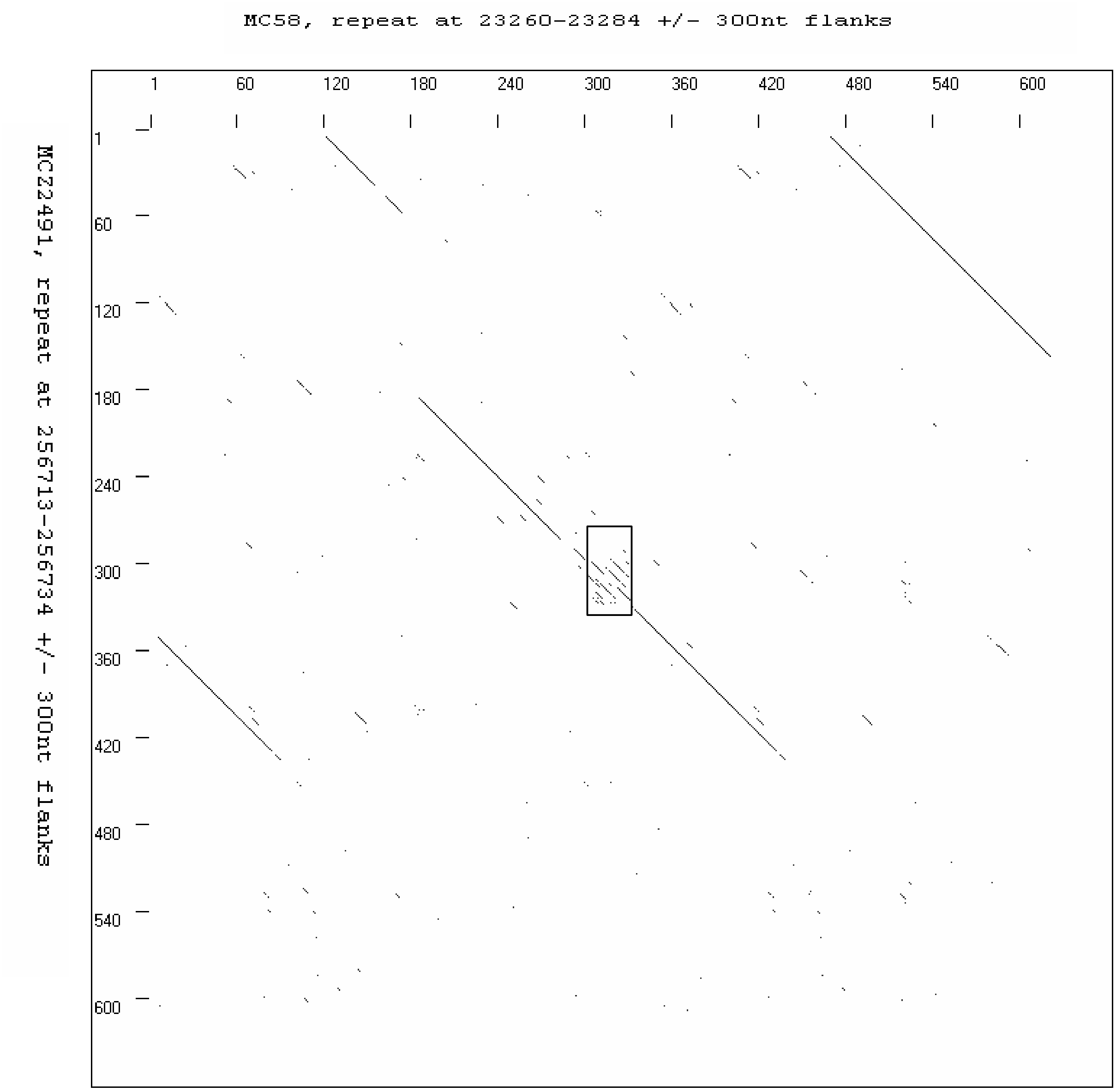

VNTRfinder: 23260-23284 253305-253409

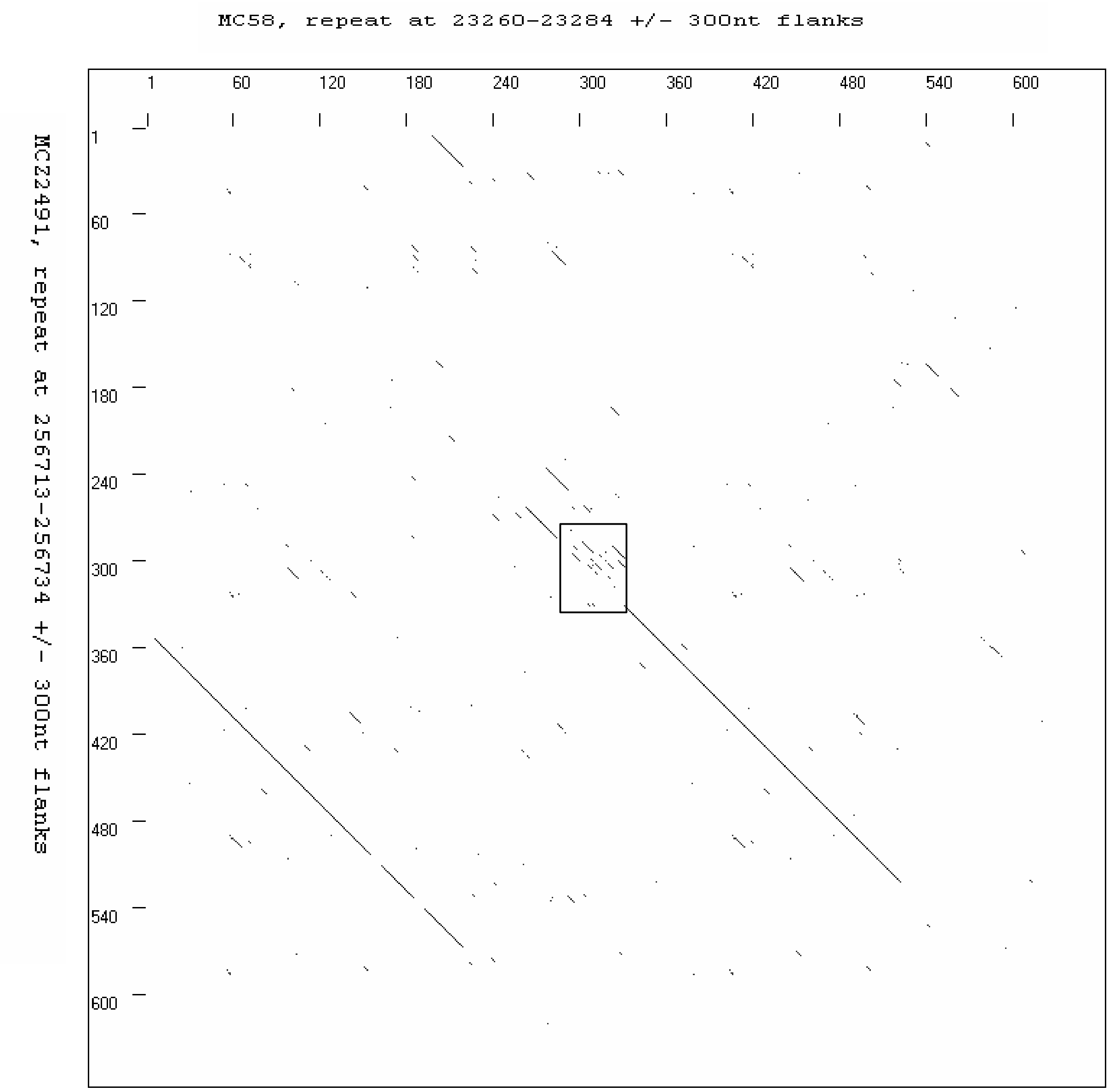

Repeat start-stop: 24341-24365
Unit: TGTT
Tandem array: TGTTTGTTTGACTGTTTTATTTTTT
Denoeud report variant of 153
VNTRfinder report variant of 0

Denoeud: 24341-24365 versus 257316-257493

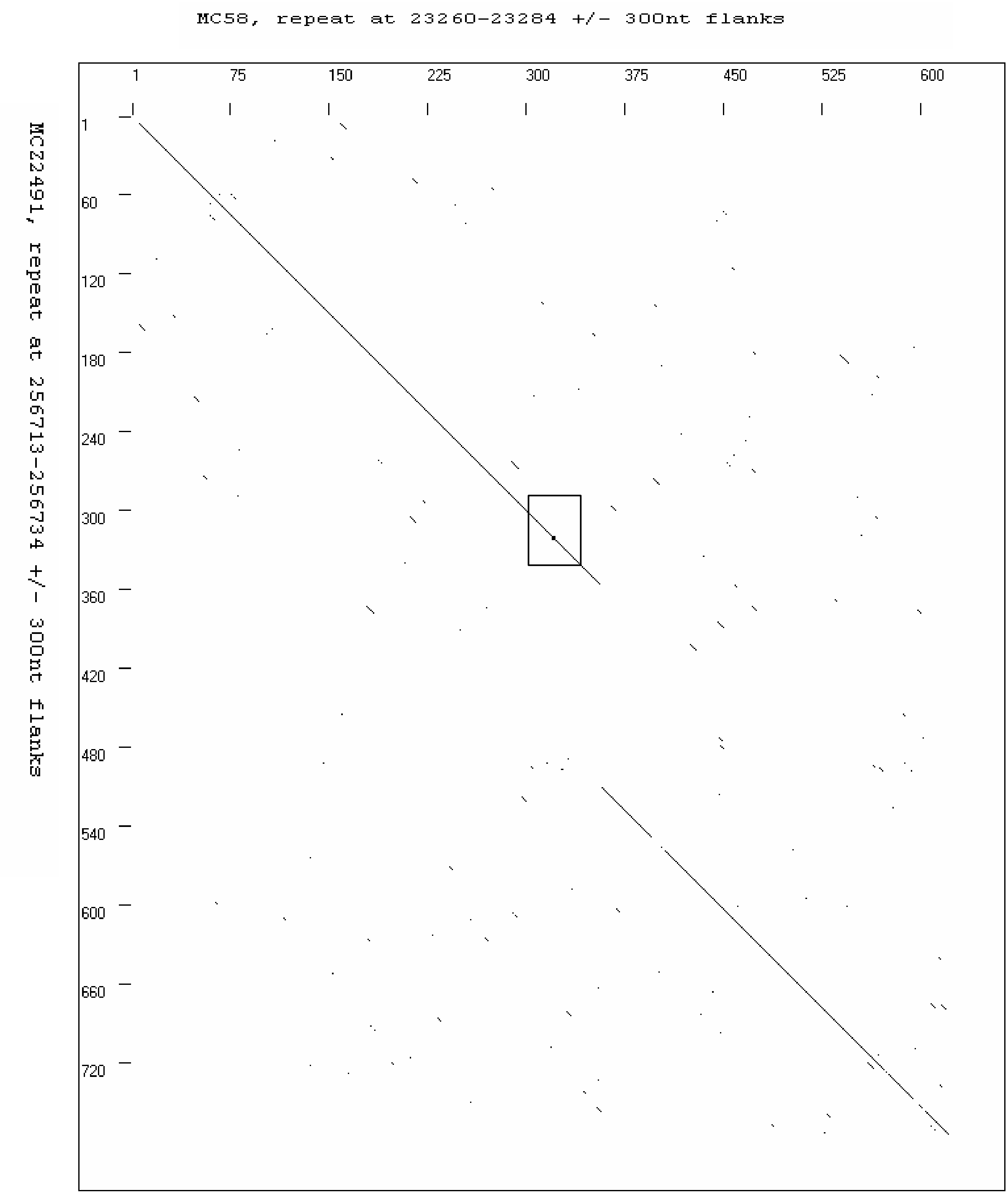

VNTRfinder: 24341-24365 257316-257340

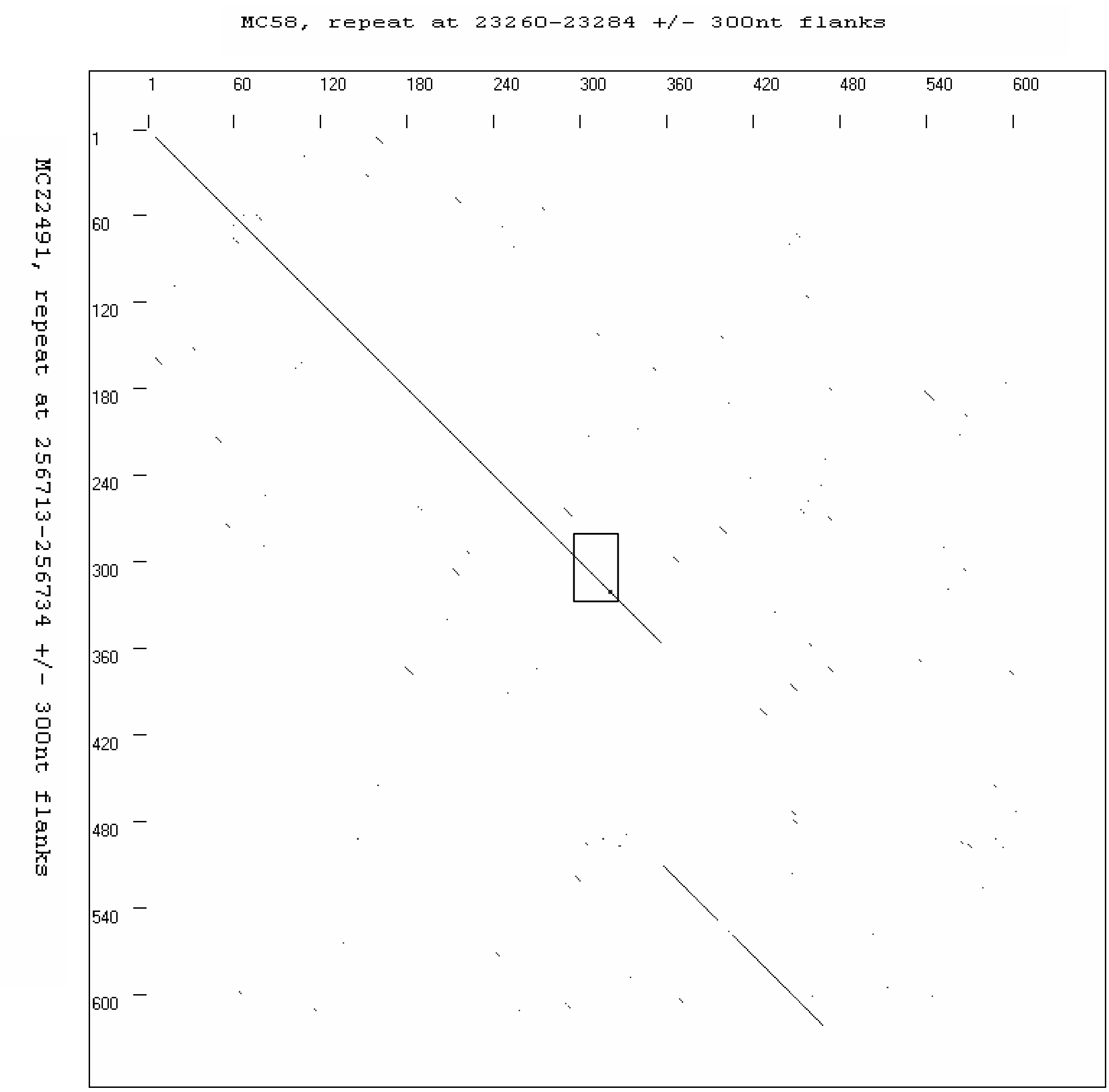

Repeat start-stop: 281145-281183
Unit: CCGCGCAATGC
Tandem array: CCGTCGCCATACCCGCGCCGAATGCCCGCGCAATGACCG
Denoeud report variant of 241
VNTRfinder report variant of 0

Denoeud: 281145-281183 versus 2163596-2163317

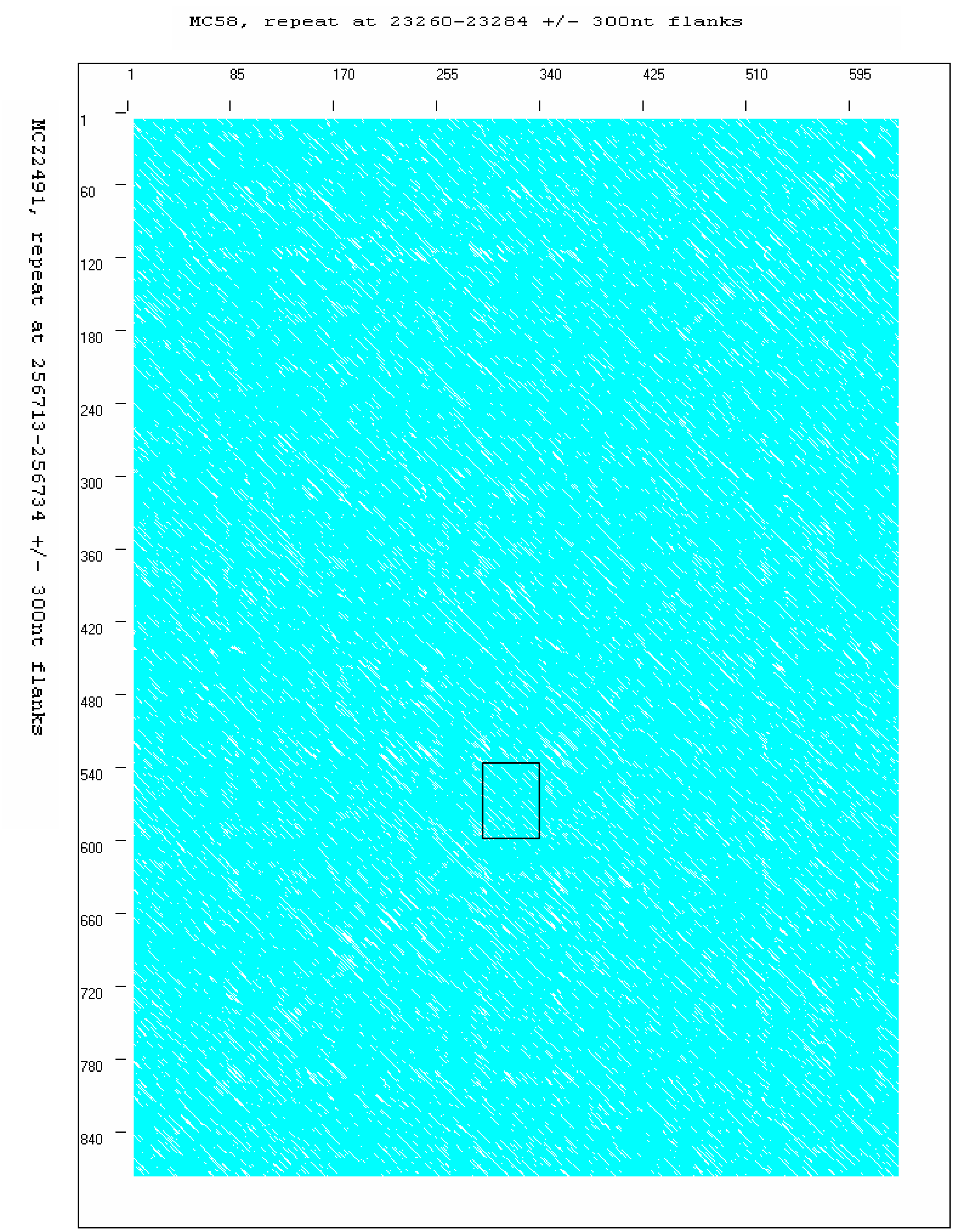

VNTRfinder: 281145-281183 2163558-2163596

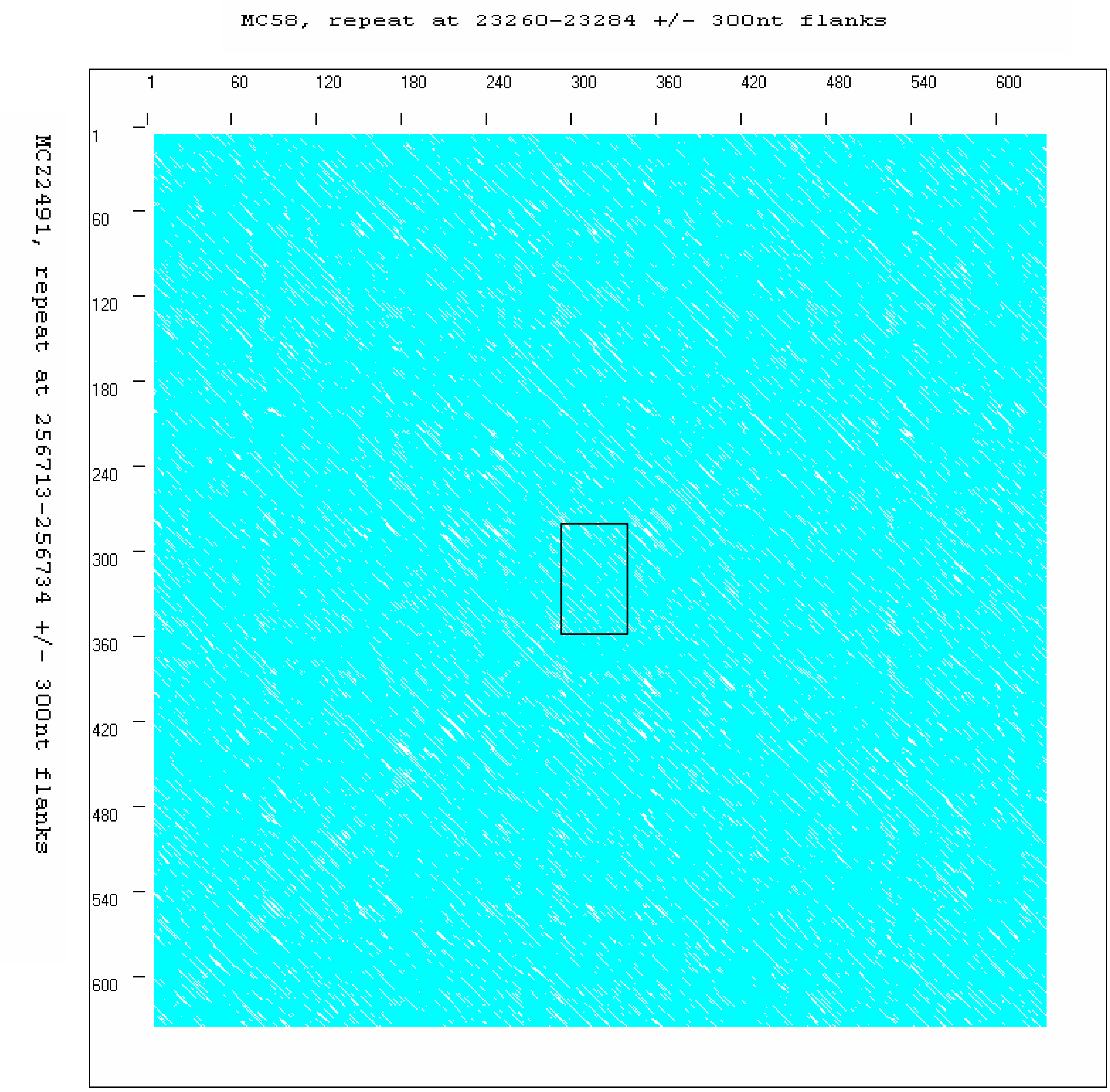

Repeat start-stop: 370747-370757
Unit: ACGGT
Tandem array: ACGGTACGGTA
Denoeud report variant of 6
VNTRfinder report variant of 0

Denoeud: 370747-370757 versus 2074675-2074671

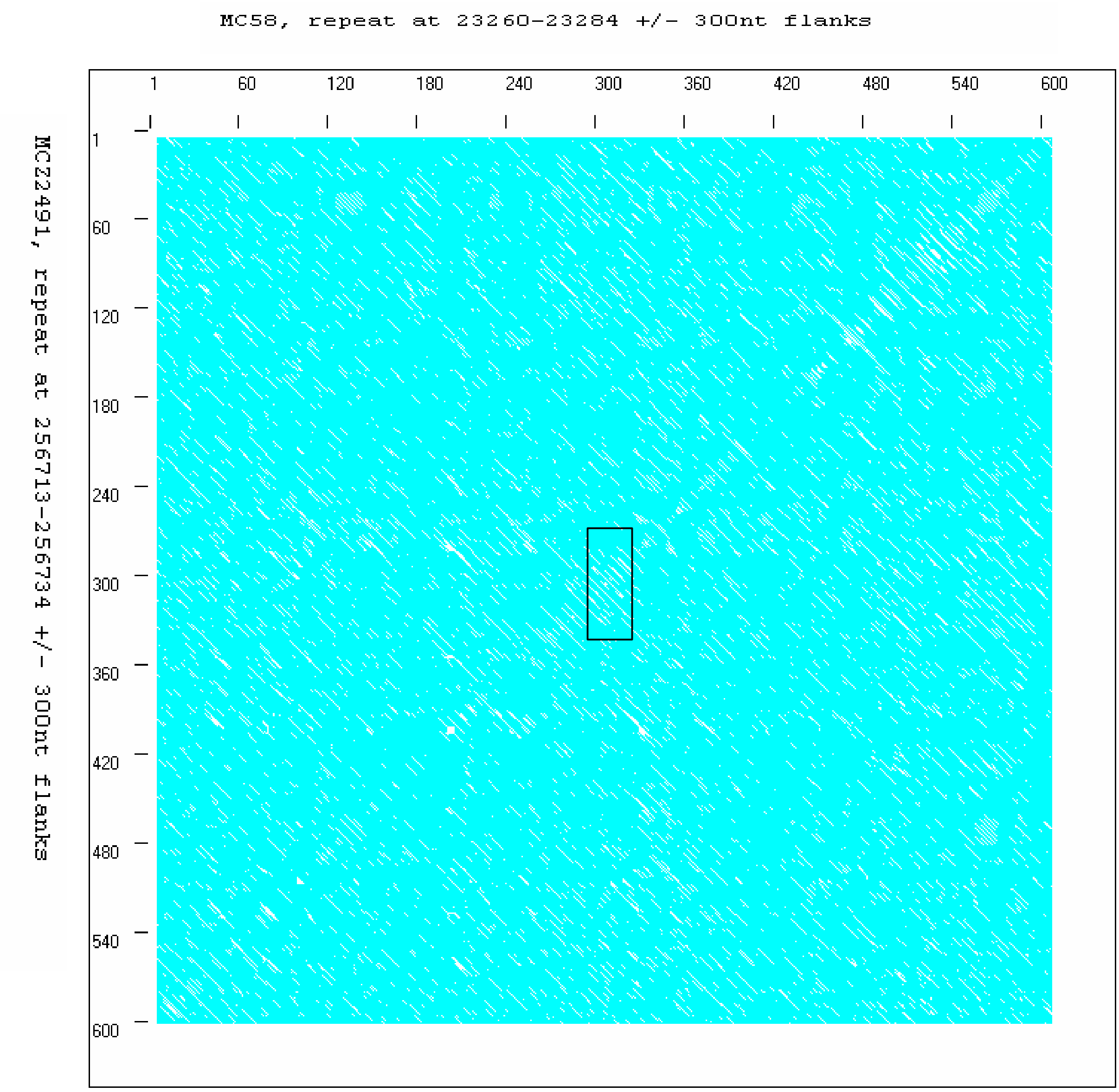

VNTRfinder: 370747-370757   2074671-2074681

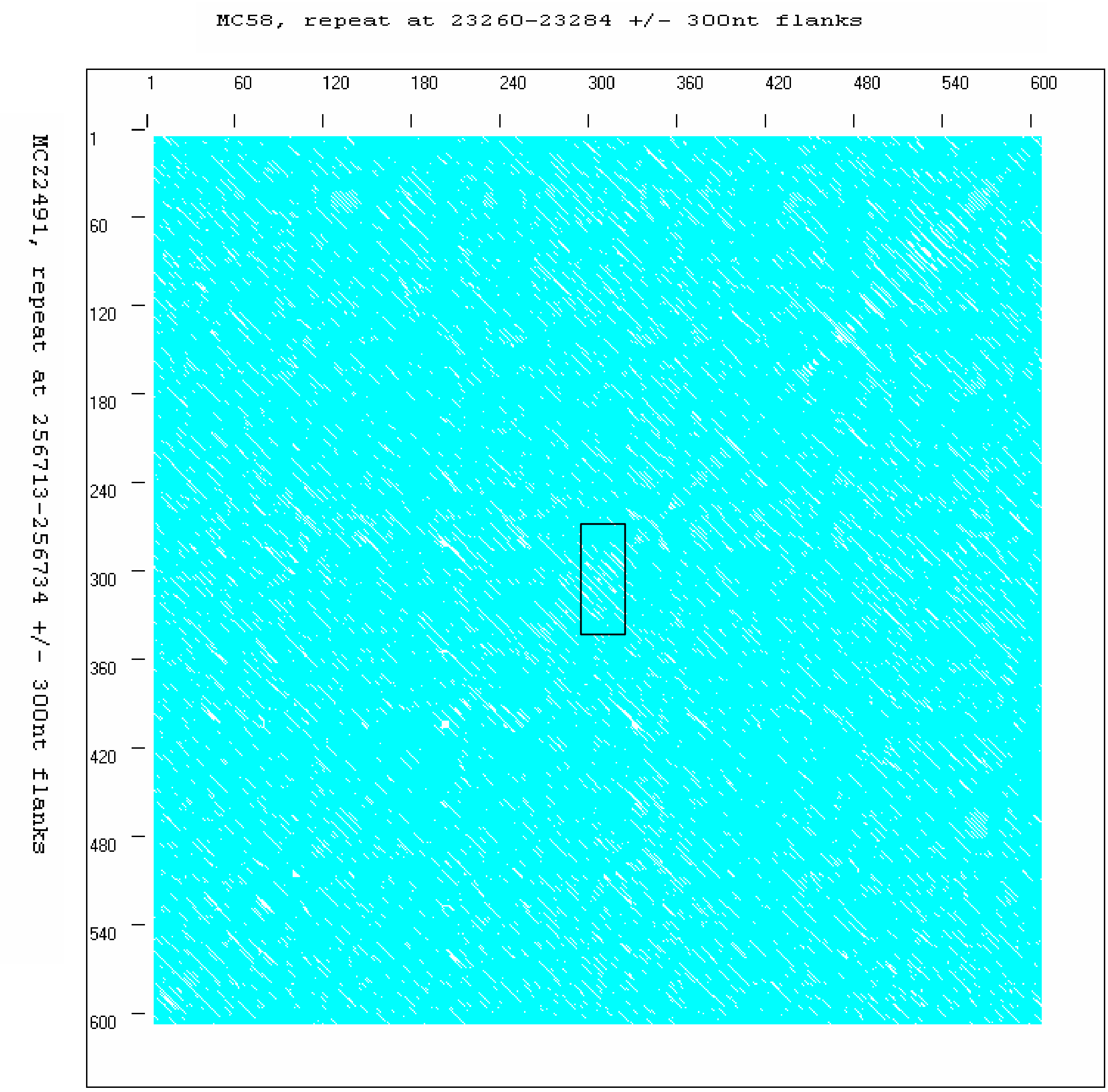

Repeat start-stop: 370778-370800
Unit: AGC
Tandem array: AGCAGCAGCTCACACAGCAGGAG
Denoeud report variant of 6
VNTRfinder report variant of 0

Denoeud: 370778-370800 versus 2074644-2074628

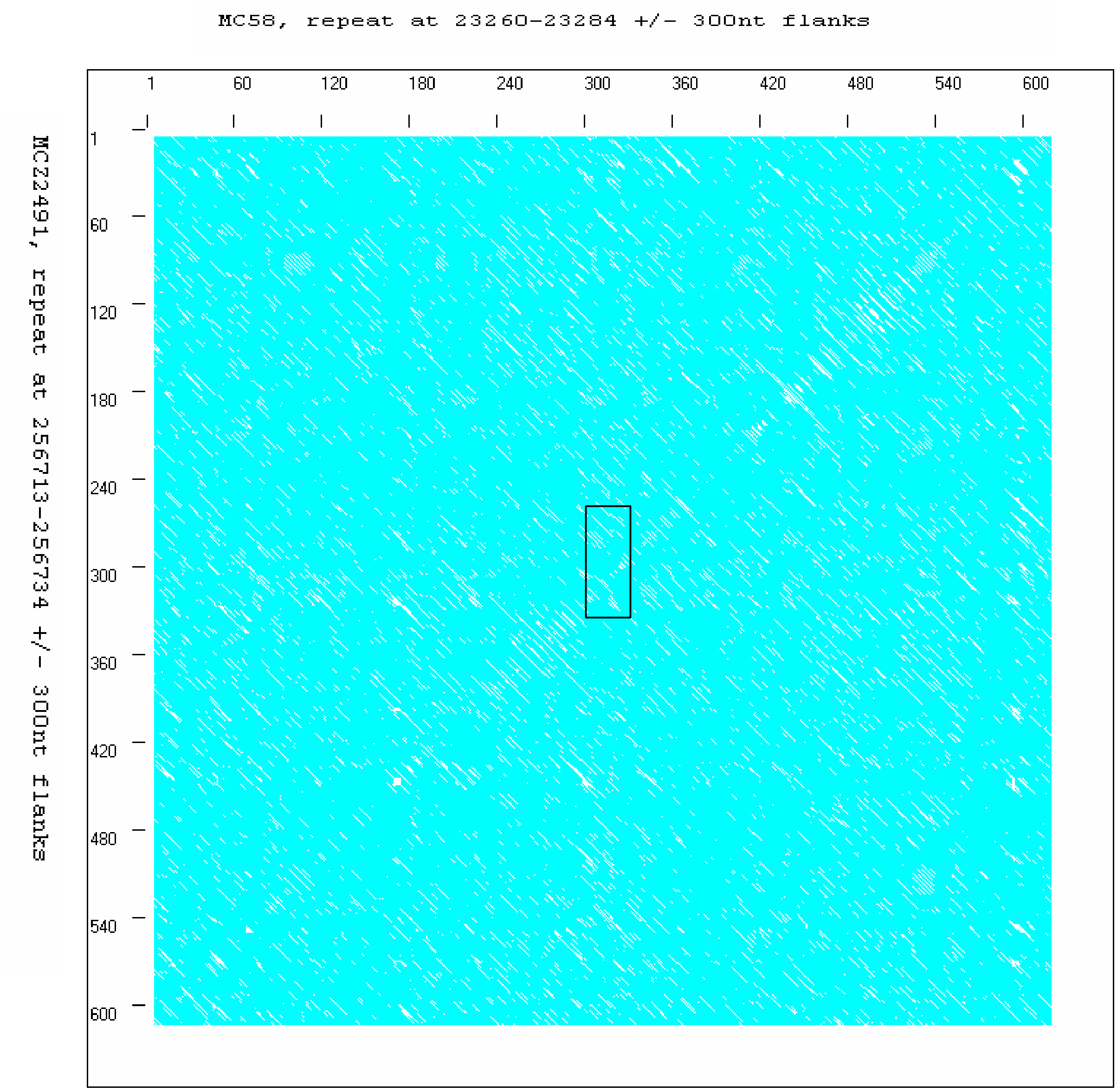

VNTRfinder: 370778-370800 2074628-2074650

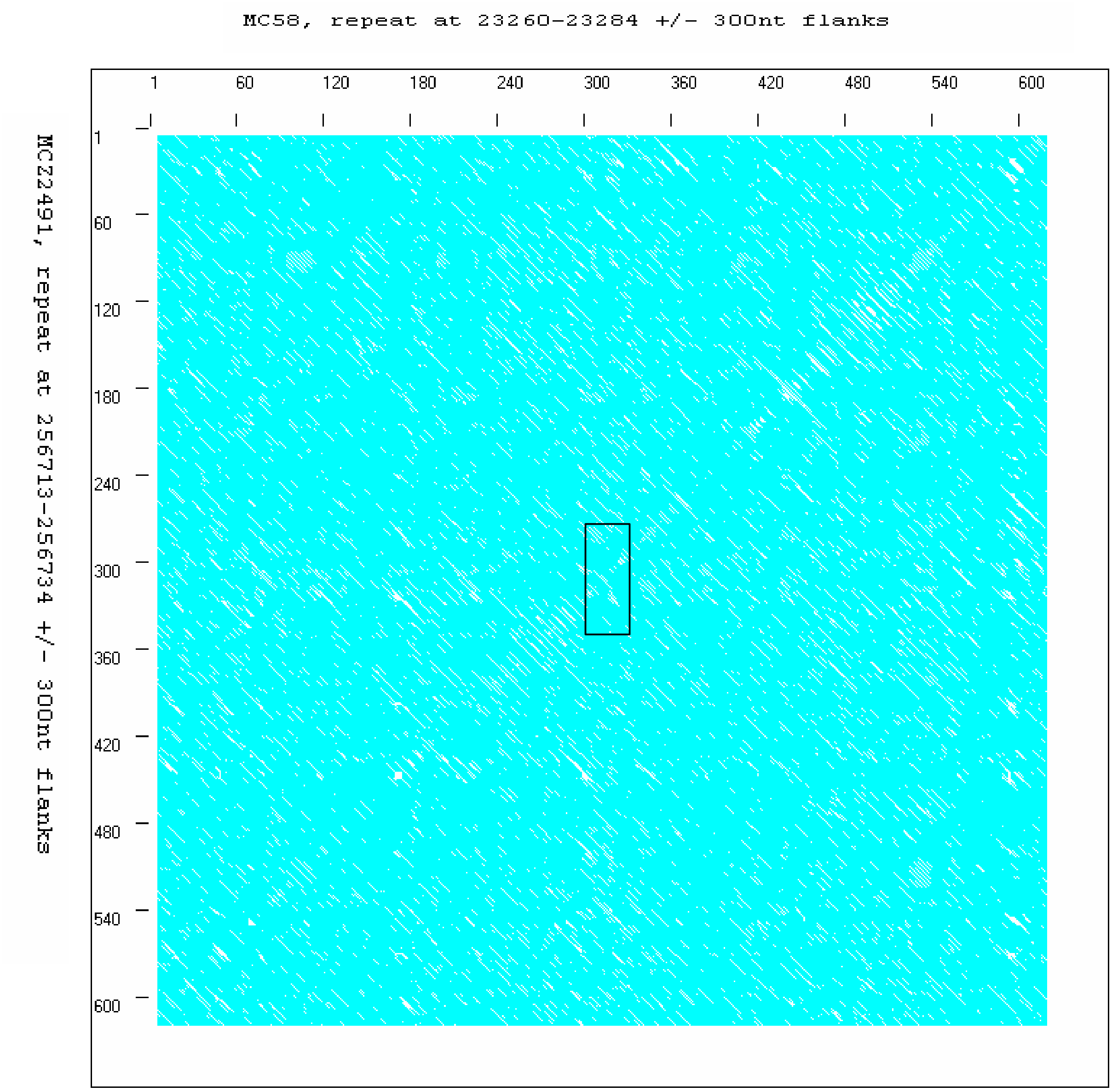

Repeat start-stop: 598226-598240
Unit: GATTT
Tandem array: GATATTGATTTGATT
Denoeud report variant of 0
VNTRfinder report variant of 1

Denoeud: 598226-598240 versus 745362-745376

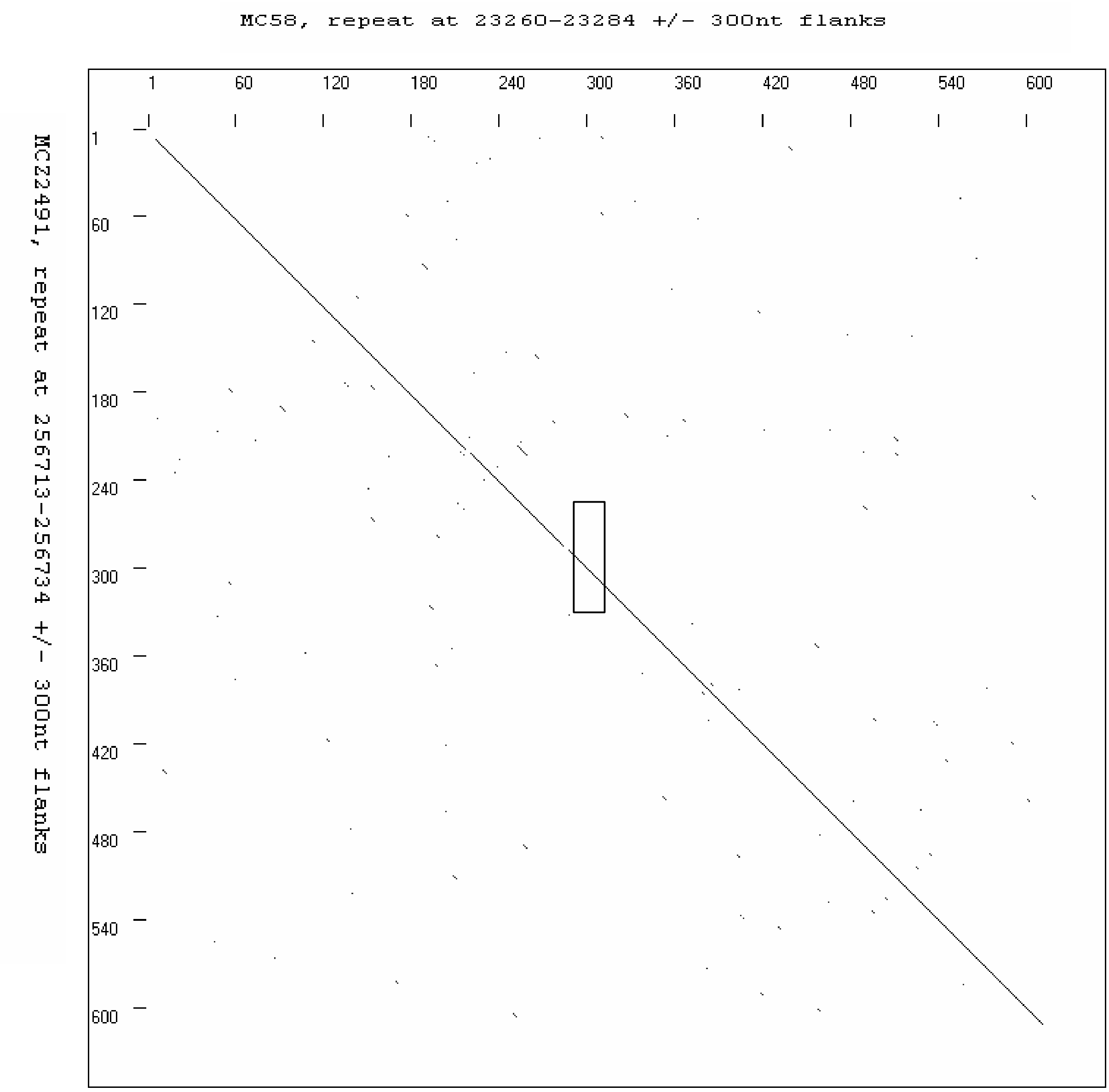

VNTRfinder: 598226-598240   745363-745376

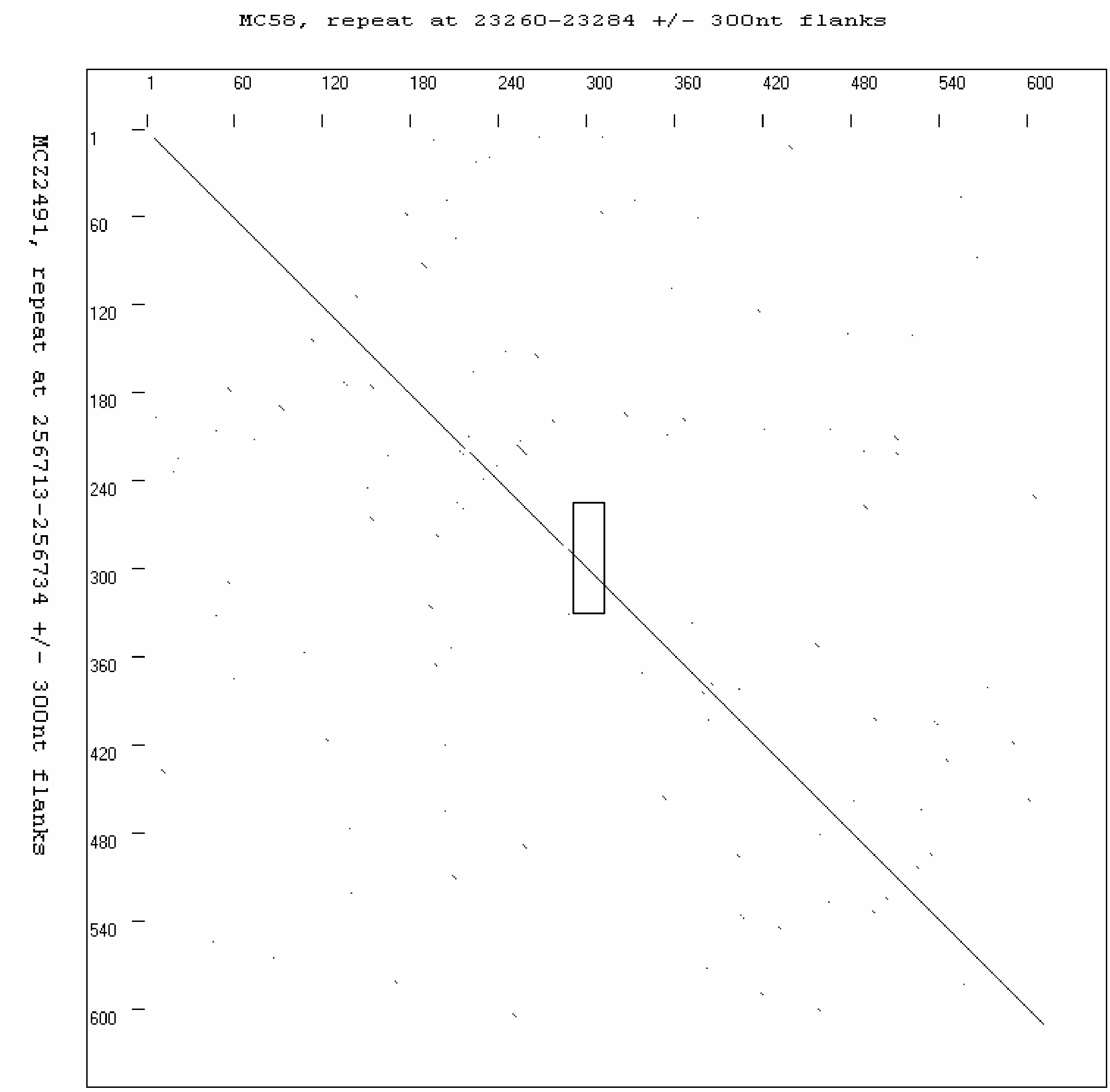

Repeat start-stop: 613994-614016
Unit: AGC
Tandem array: AGCAGCAGCTCACACAGCAGGAG
Denoeud report variant of 6
VNTRfinder report variant of 0

Denoeud: 613994-614016 2074644-2074628

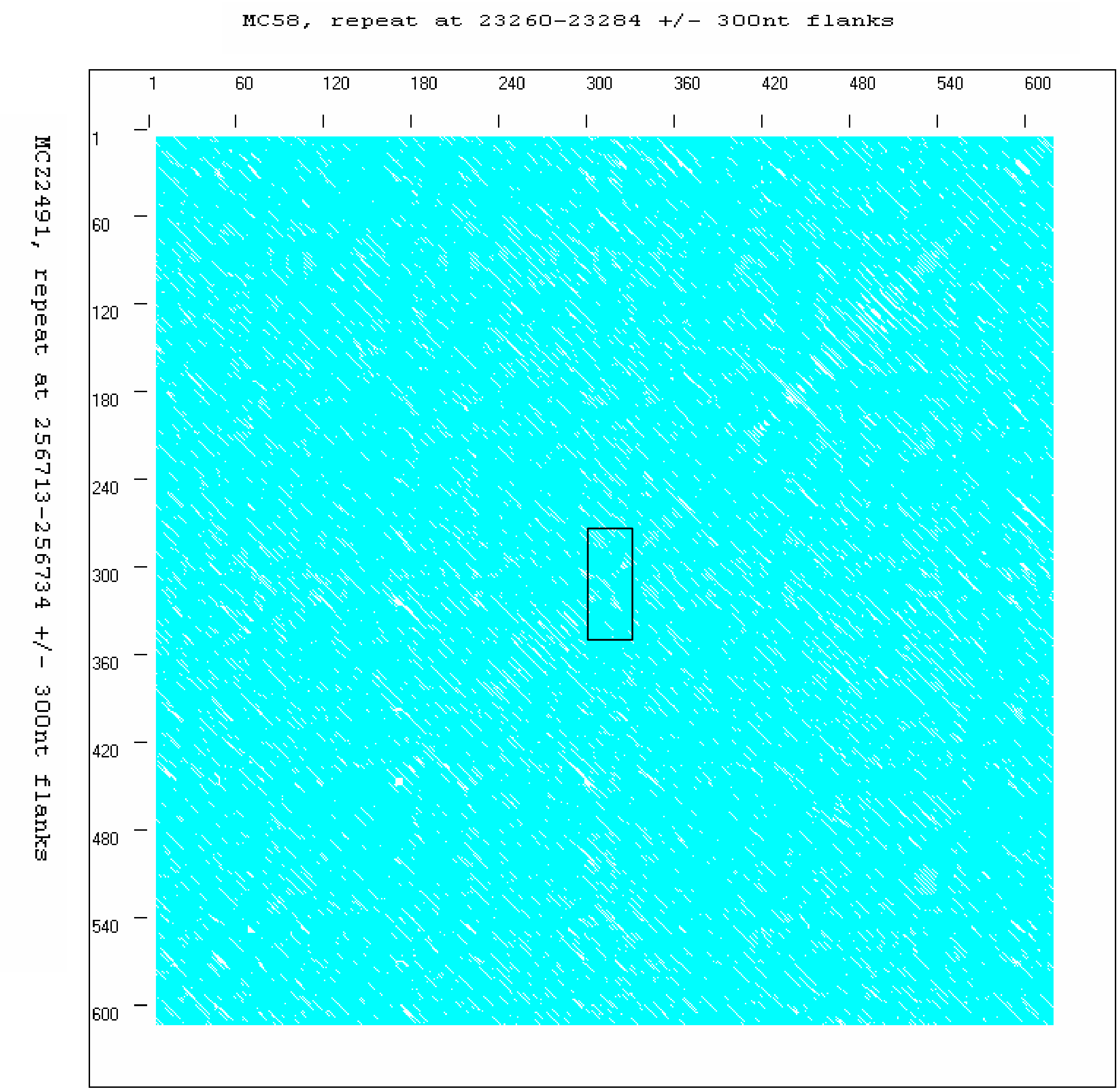

VNTRfinder: 613994-614016 2074628-2074650

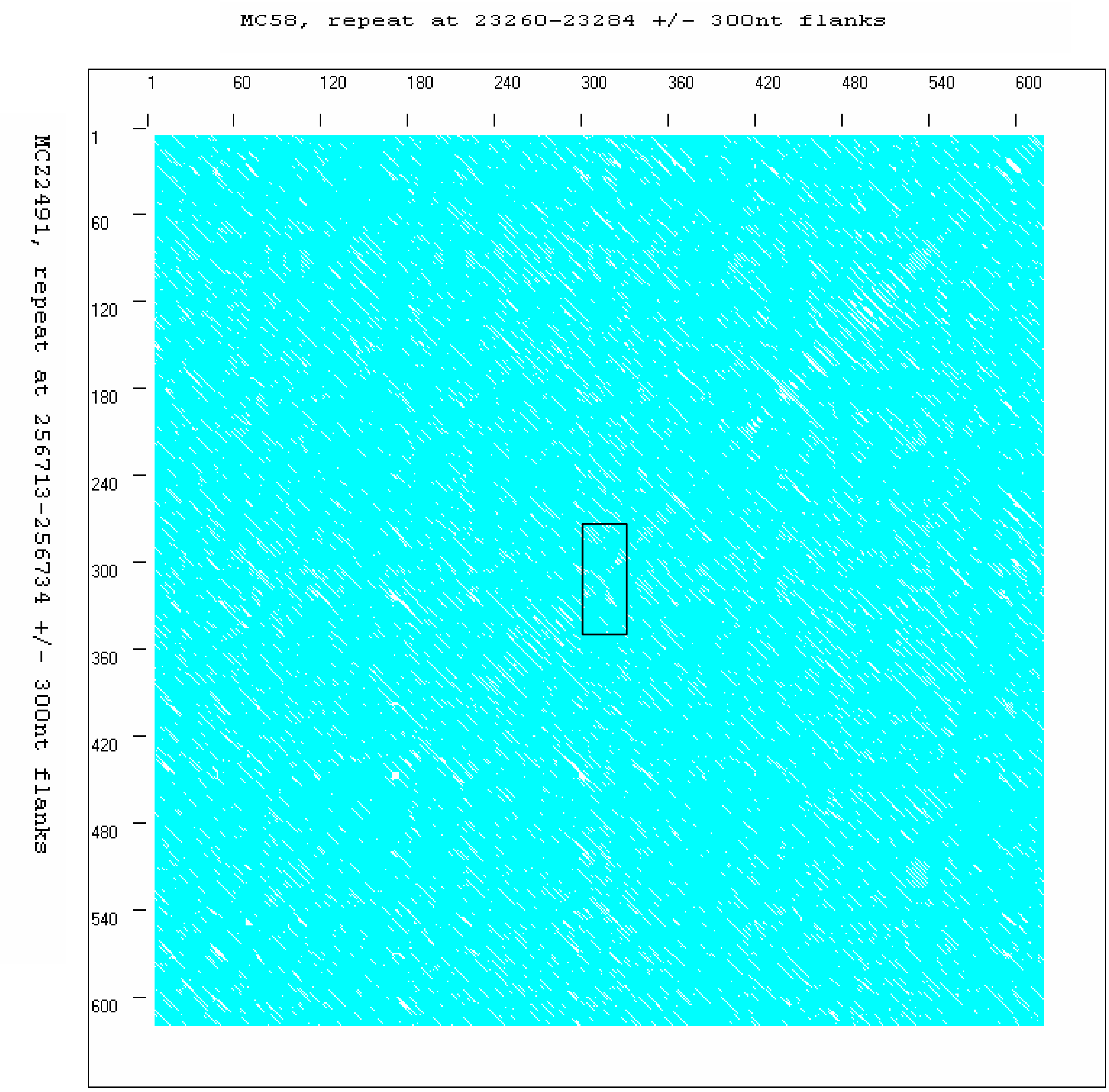

Repeat start-stop: 794146-794168
Unit: CATTGTTGTT
Tandem array: CATTGTGTTCATTGTTGTTCCTT
Denoeud report variant of 1
VNTRfinder report variant of 0

Denoeud: 794146-794168 941942-941963

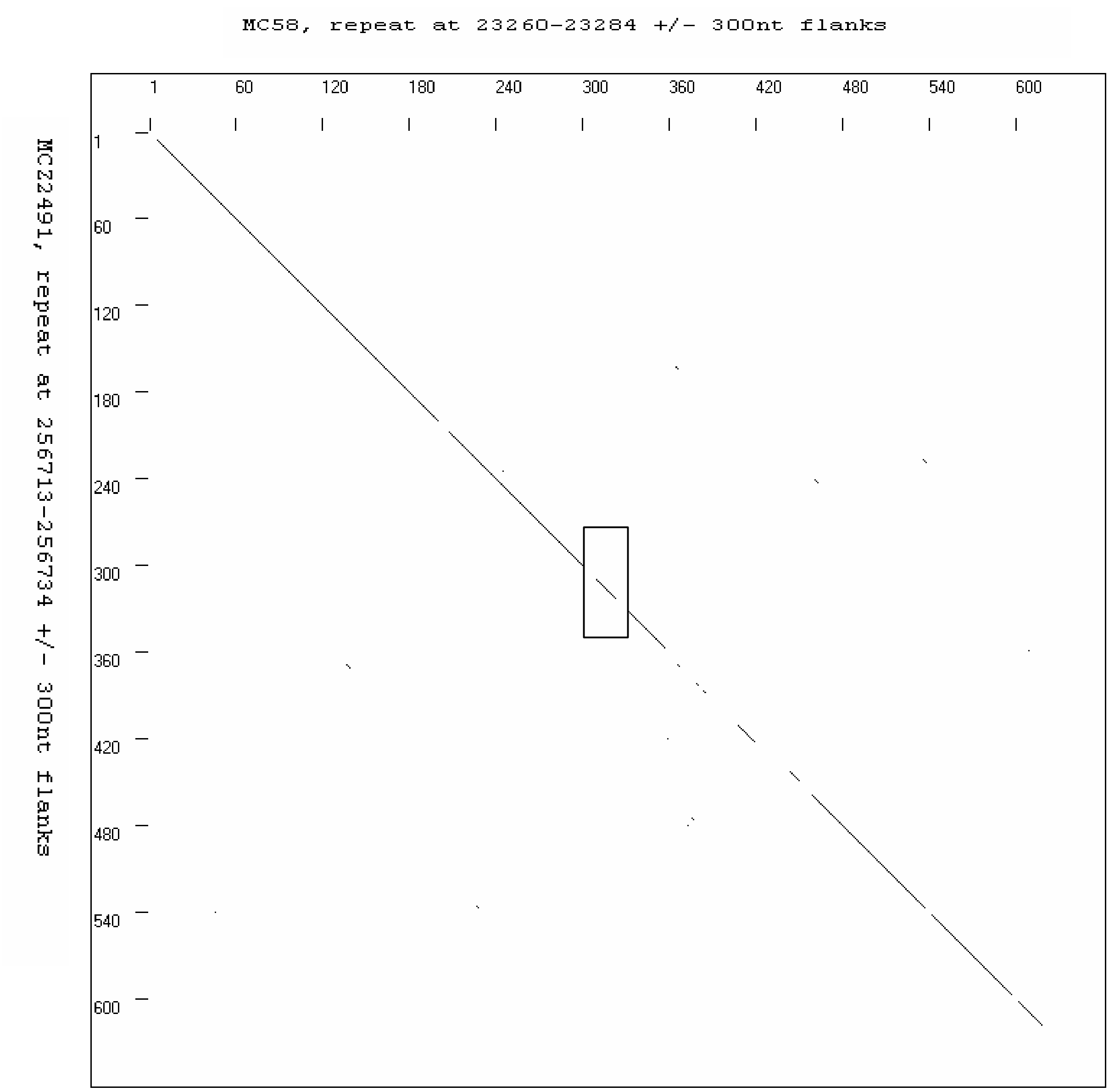

VNTRfinder: 794146-794168   941942-941964

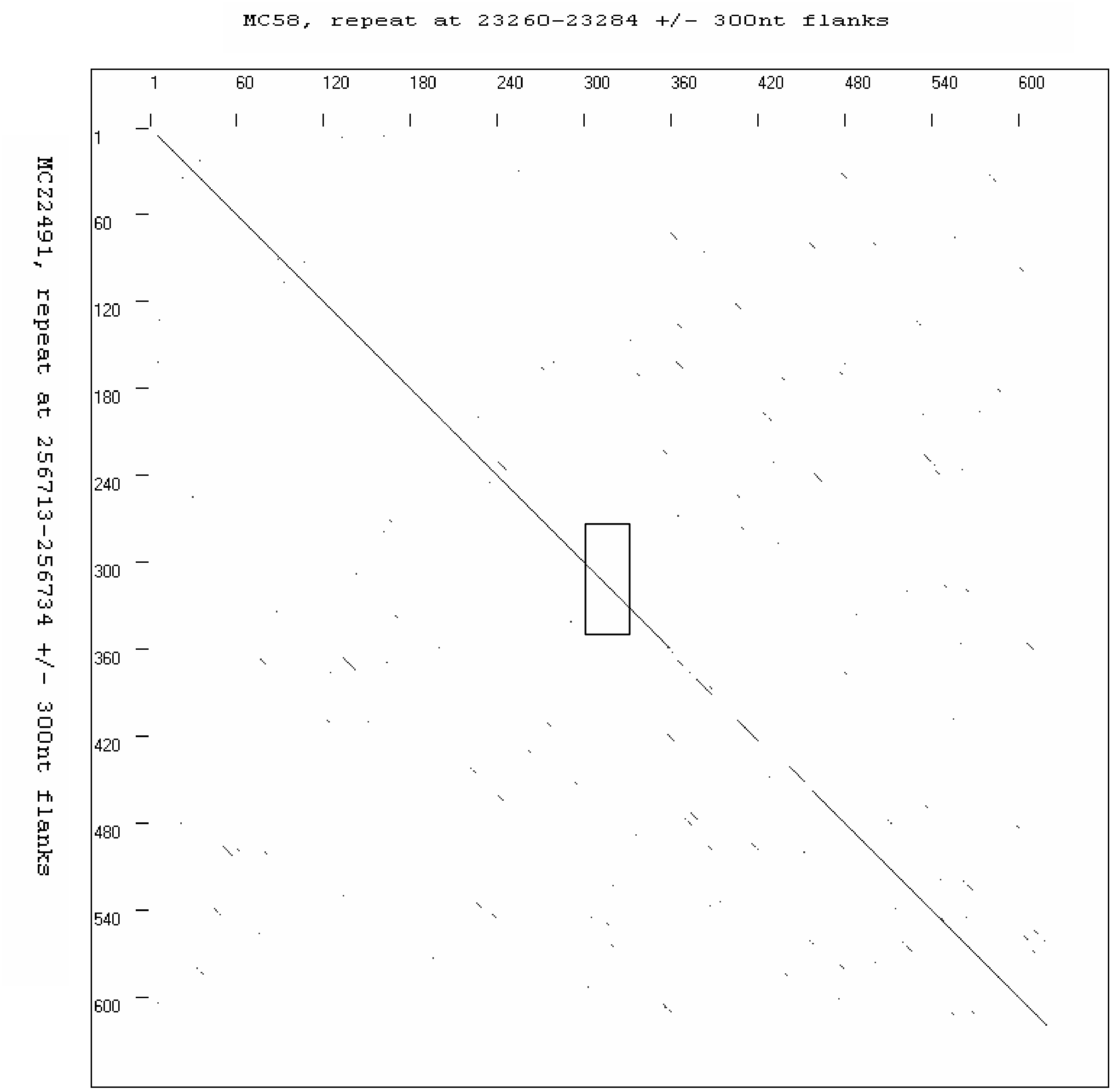

Repeat start-stop: 1340997-1341015
Unit: TGGCGGCTG
Tandem array: TGGCGGCTGTGGCTGCTGT
Denoeud report variant of 15
VNTRfinder report variant of 0

Denoeud: 1340997-1341015 1432907-1432940

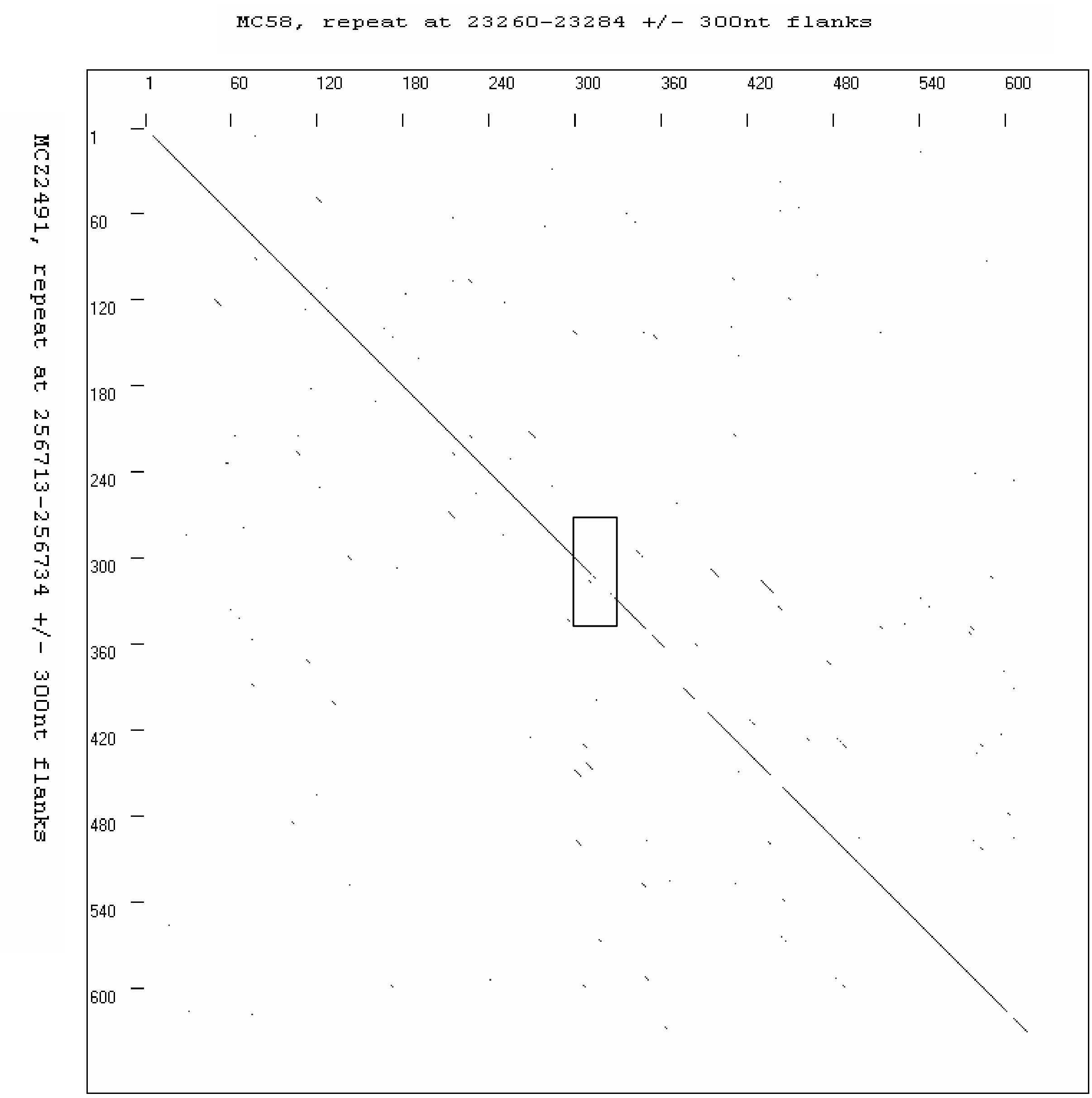

VNTRfinder: 1340997-1341015 1432907-1432925

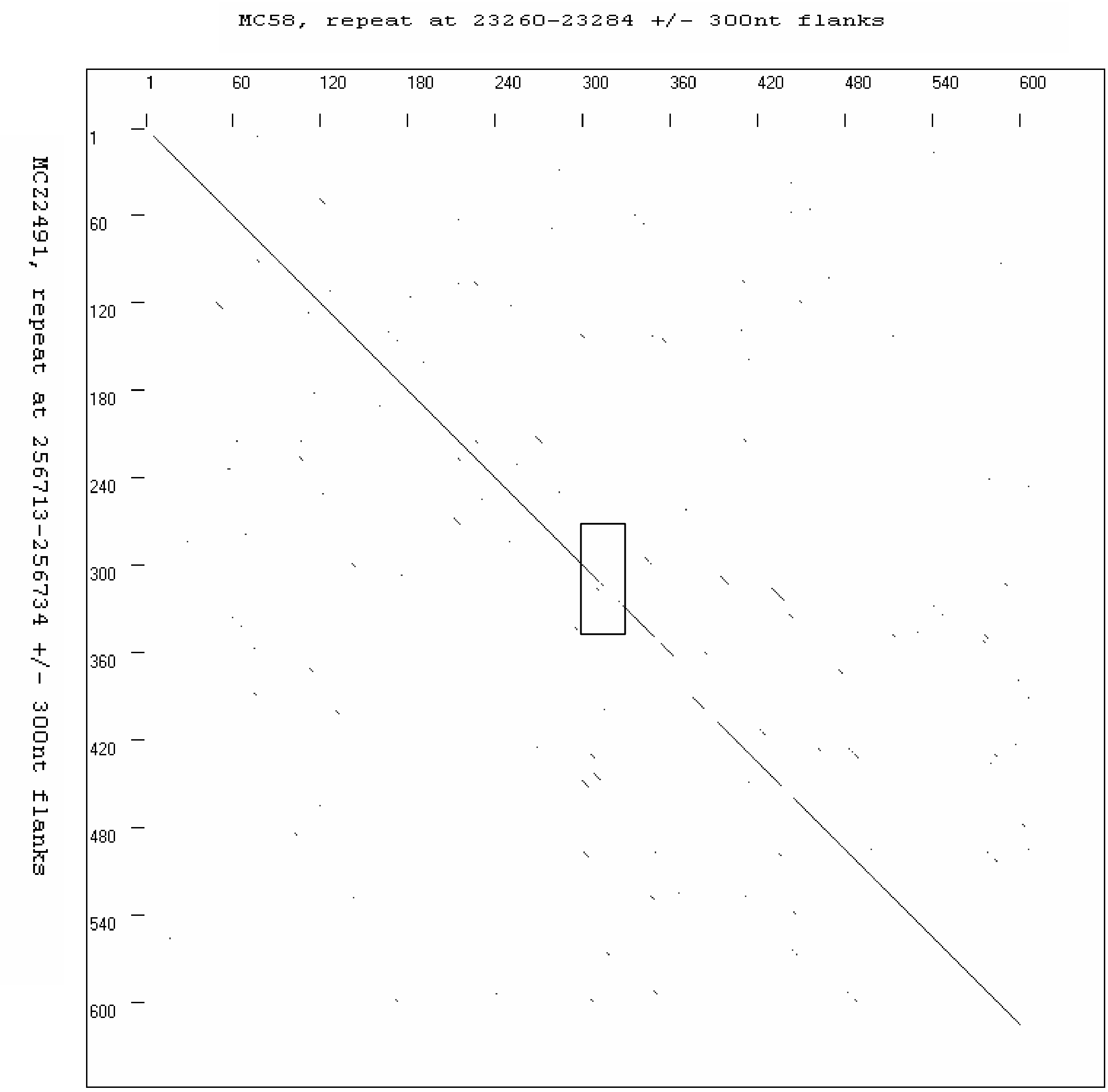

**Repeat start-stop: 1443509-1443531**<br>**Unit: AGC**<br>**Tandem array: AGCAGCAGCTCACACAGCAGGAG**<br>**Denoeud report variant of 6**<br>**VNTRfinder report variant of 0**

```
Denoeud:    1443509-1443531   2074644-2074628
VNTRfinder: 1443509-1443531   2074628-2074650
```

(Same problematic sequence as before)

**Repeat start-stop: 1452043-1452078**<br>**Unit: TTTG**<br>**Tandem array: TTTGCTCATTTTTTTCTTTCAATGTTATTTTGTTTG**<br>**Denoeud report variant of 4**<br>**VNTRfinder report variant of 0**

```
Denoeud:    1452043-1452078   1535314-1535353
```

MC58, repeat at 23260-23284 +/- 300nt flanks

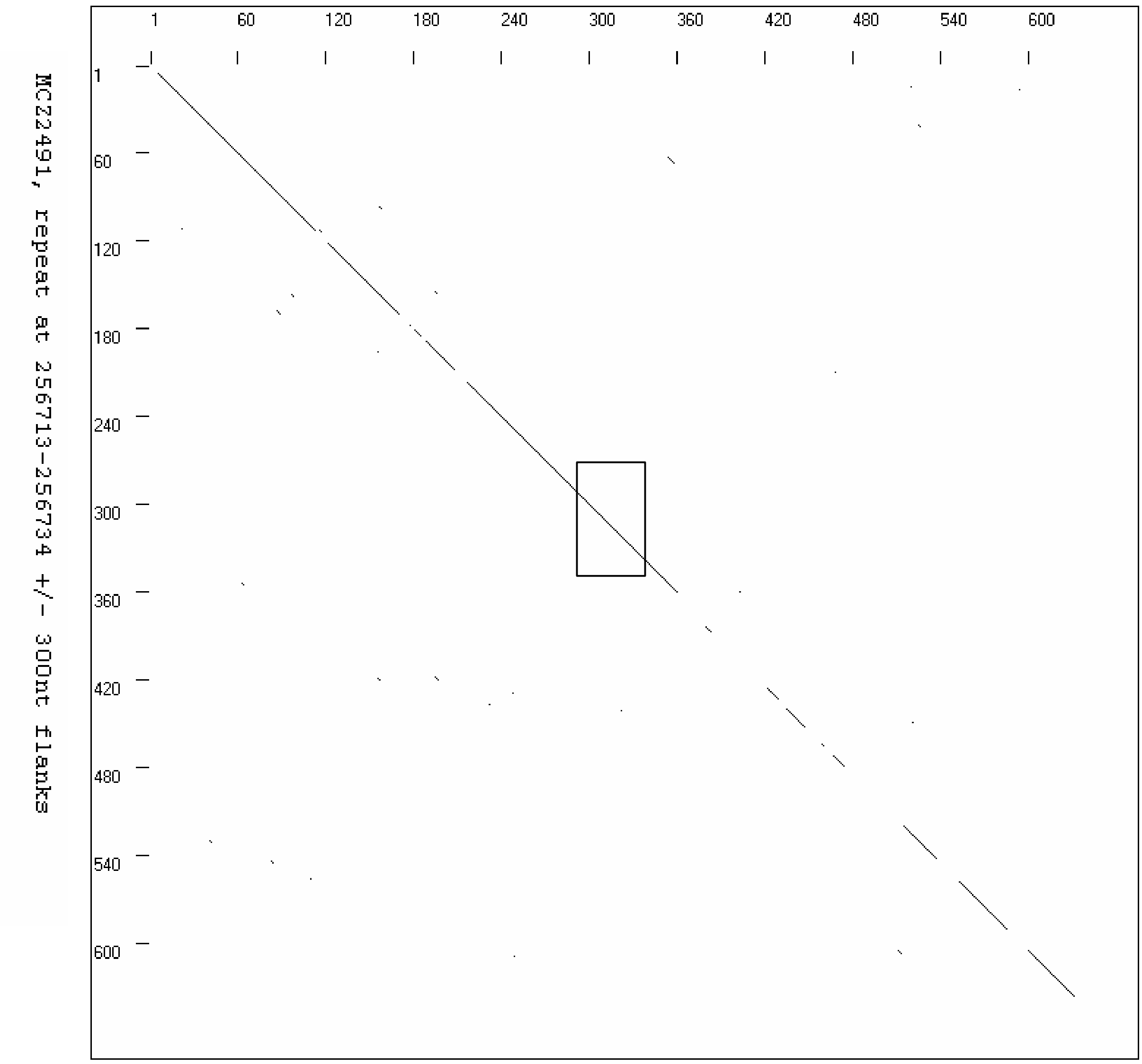

VNTRfinder: 1452043-1452078   1535314-1535349

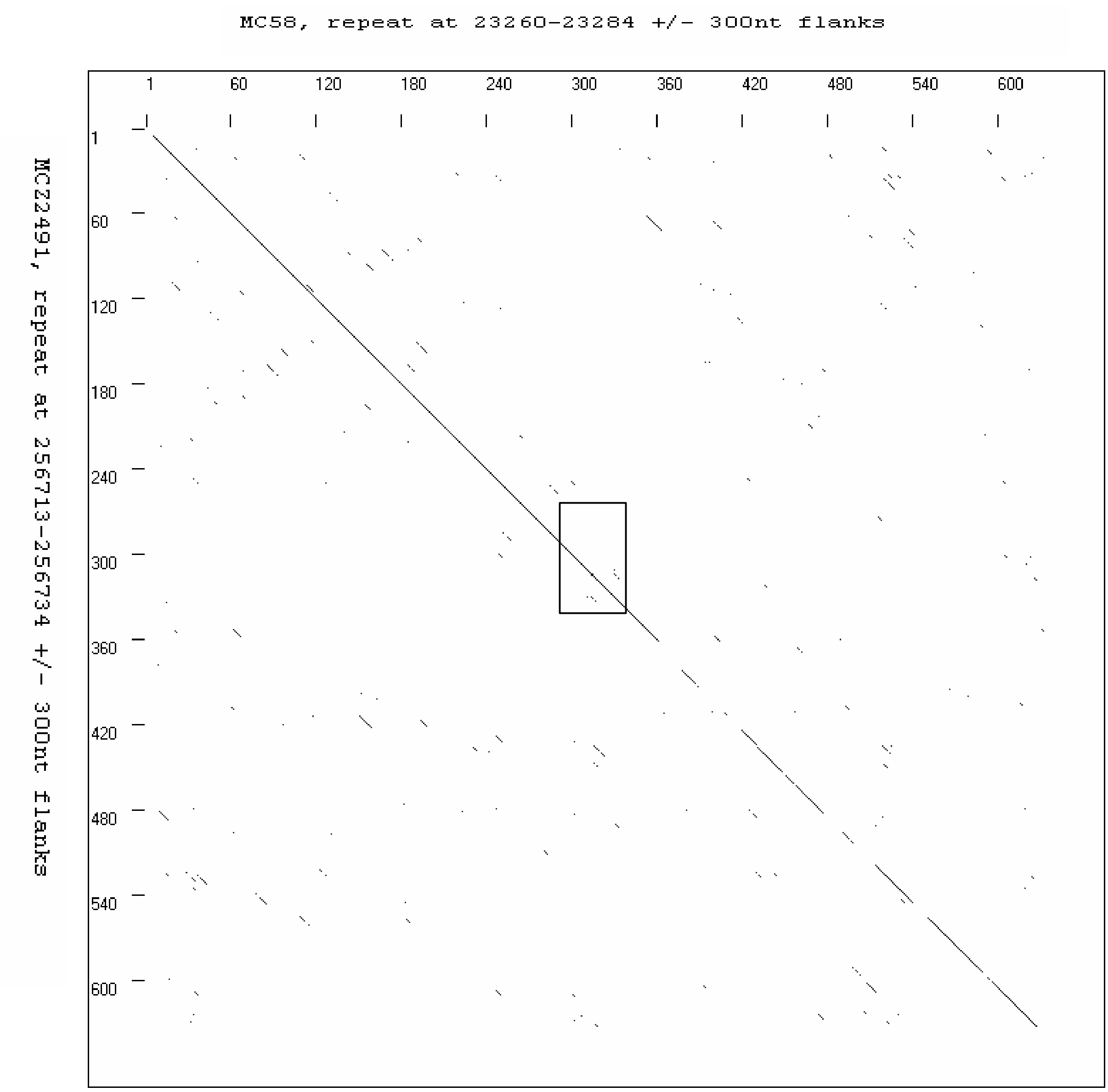

Repeat start-stop: 1487085-1487267
Unit:CCGTCATTCCCGCGAACGCGGGAATCTAGTAACCGAAAAACCACAGGAATCTATCGGAAAAAACAGAAACCCCCGA
(75mer)
Tandem array: …
Denoeud report variant of 0
VNTRfinder report variant of 3

Denoeud: 1487085-1487267 1573652-1573834

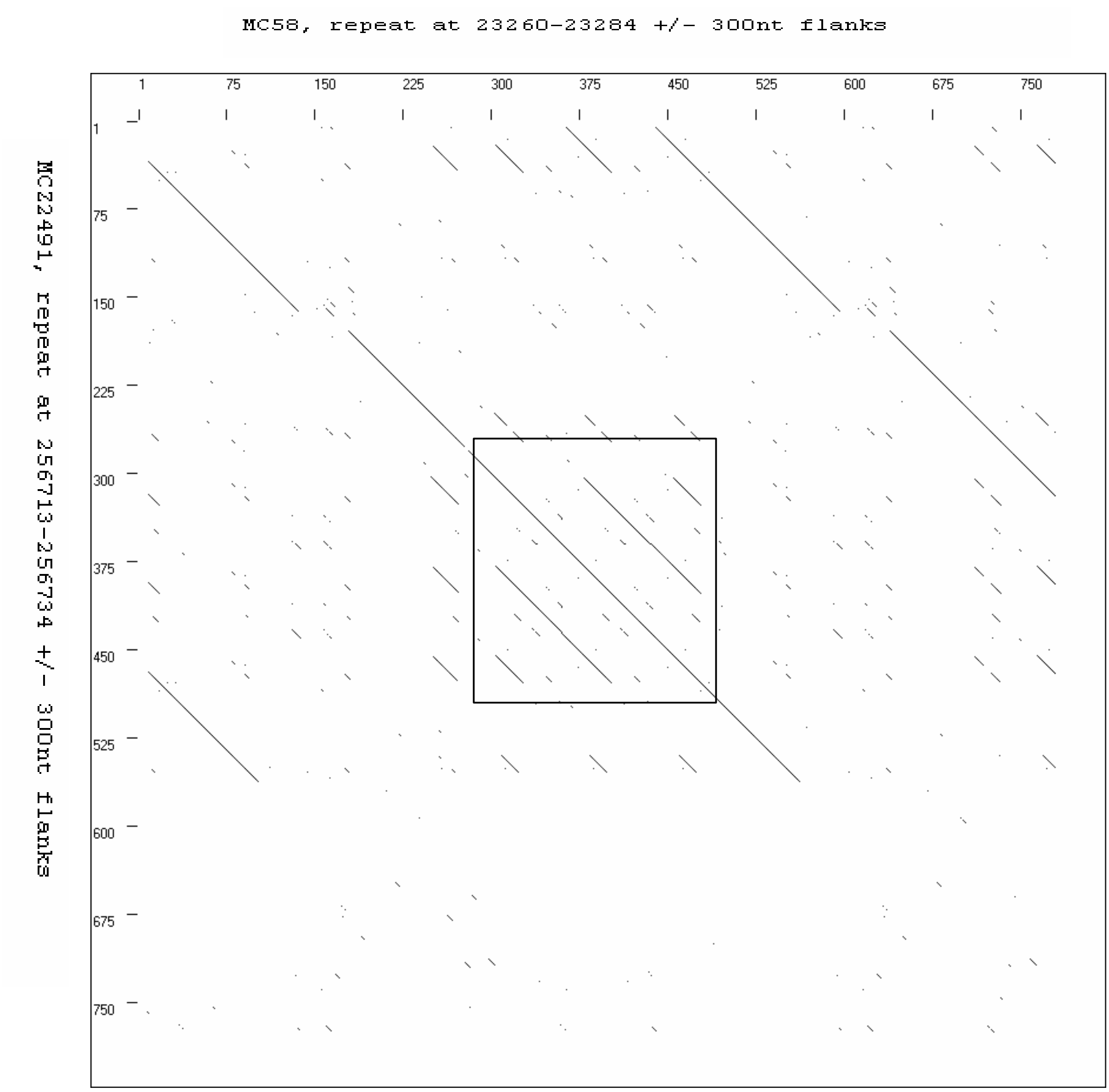

VNTRfinder: 1487085-1487267   1573215-1573400

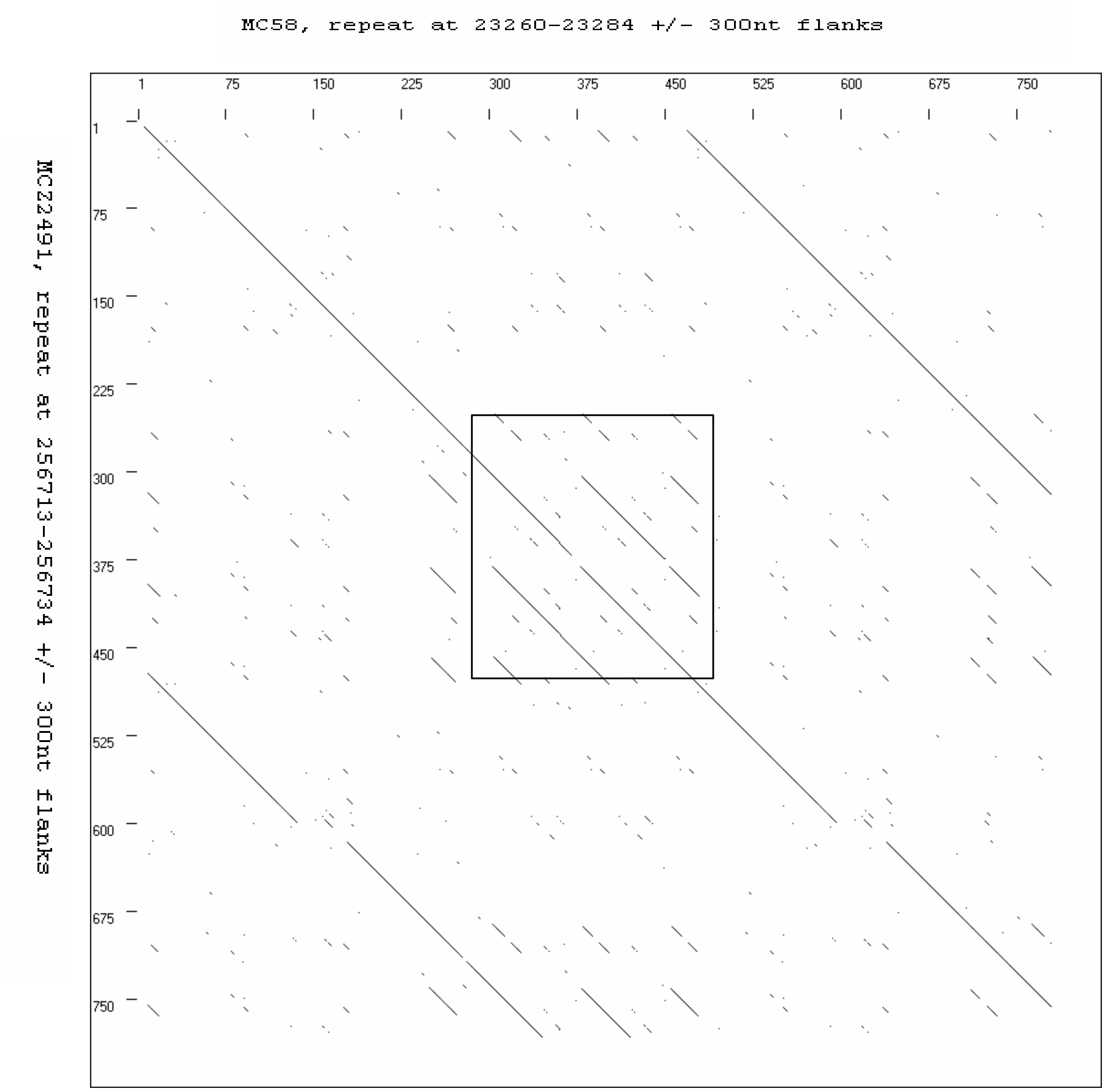

Repeat start-stop: 1527328-1527375
Unit: GTTTTCACCC
Tandem array:
GTTTTCAGCCGGTCACAGGCGGTTTTCACCTGTTTGCGCGCGATTTCA
Denoeud report variant of 5
VNTRfinder report variant of 0

Denoeud: 1527328-1527375 1612372-1612414

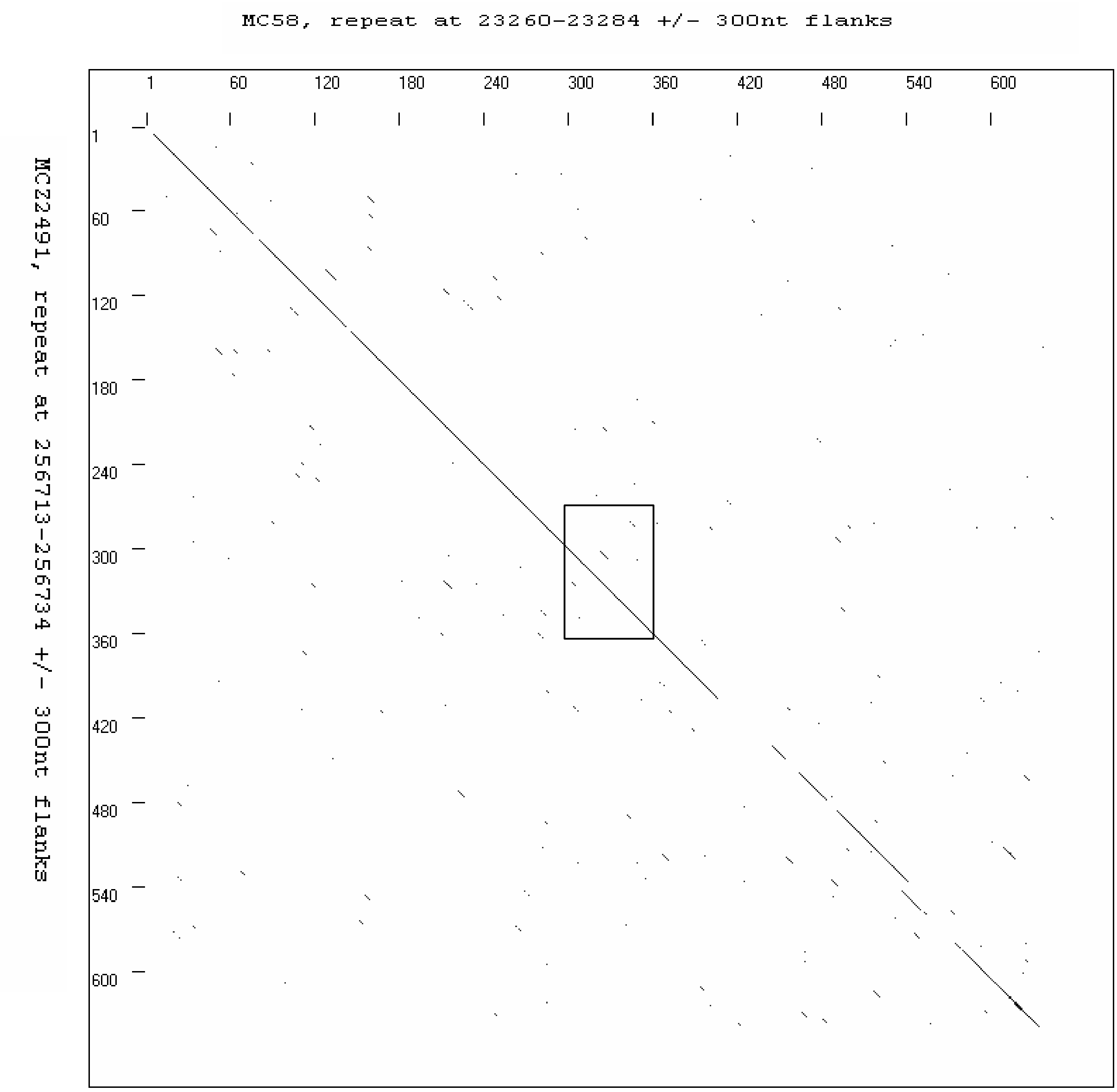

VNTRfinder: 1527328-1527375   1612372-1612419

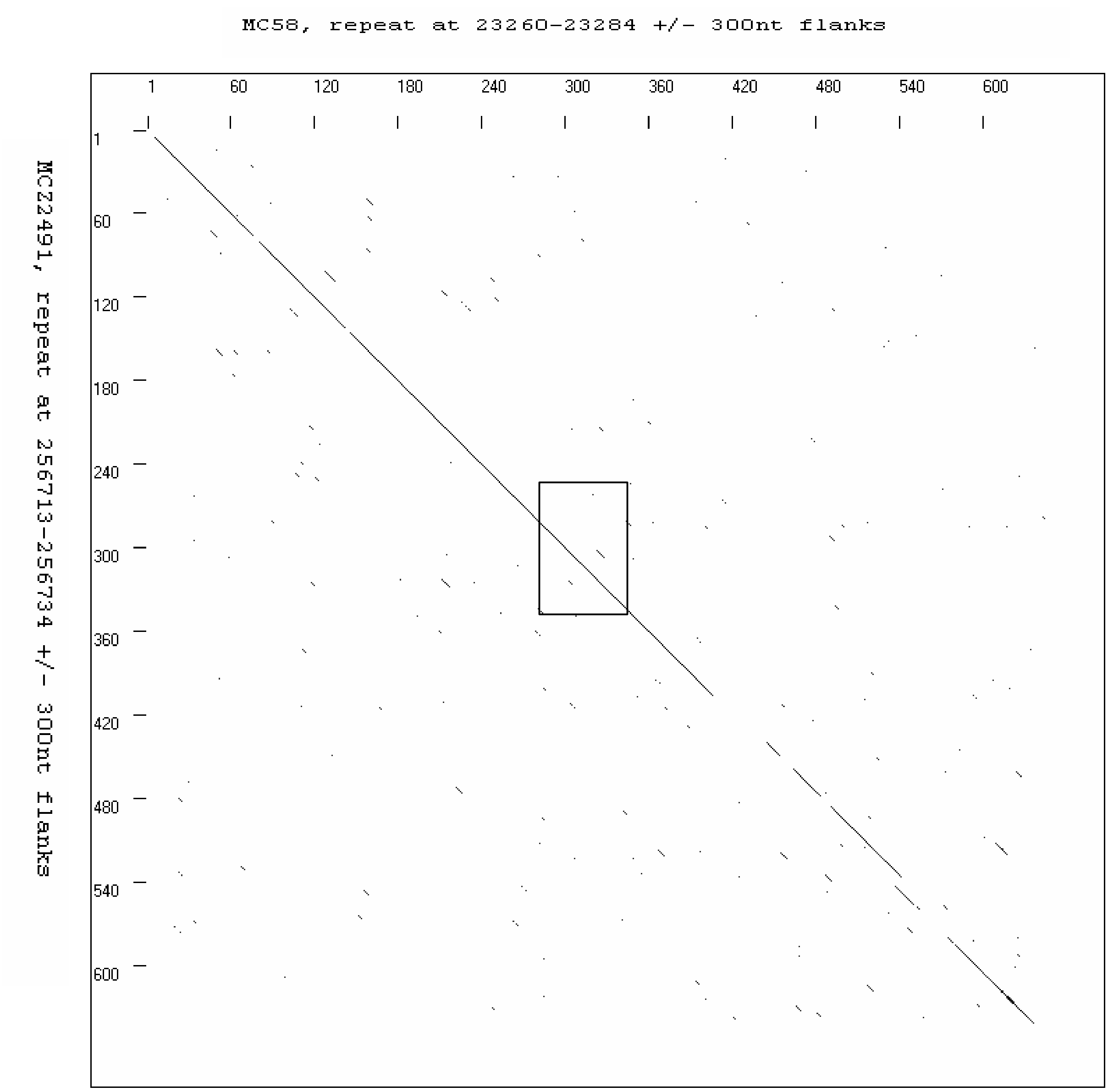

Repeat start-stop: 1604964-1605008
Unit: AATCCCGATGCA
Tandem array:
AATCCCGAACCCGACCCCGATTTGAATCCCGATGCAAATCCCGAT
Denoeud report variant of 18
VNTRfinder report variant of 0

Denoeud: 1604964-1605008 1741676-1741702

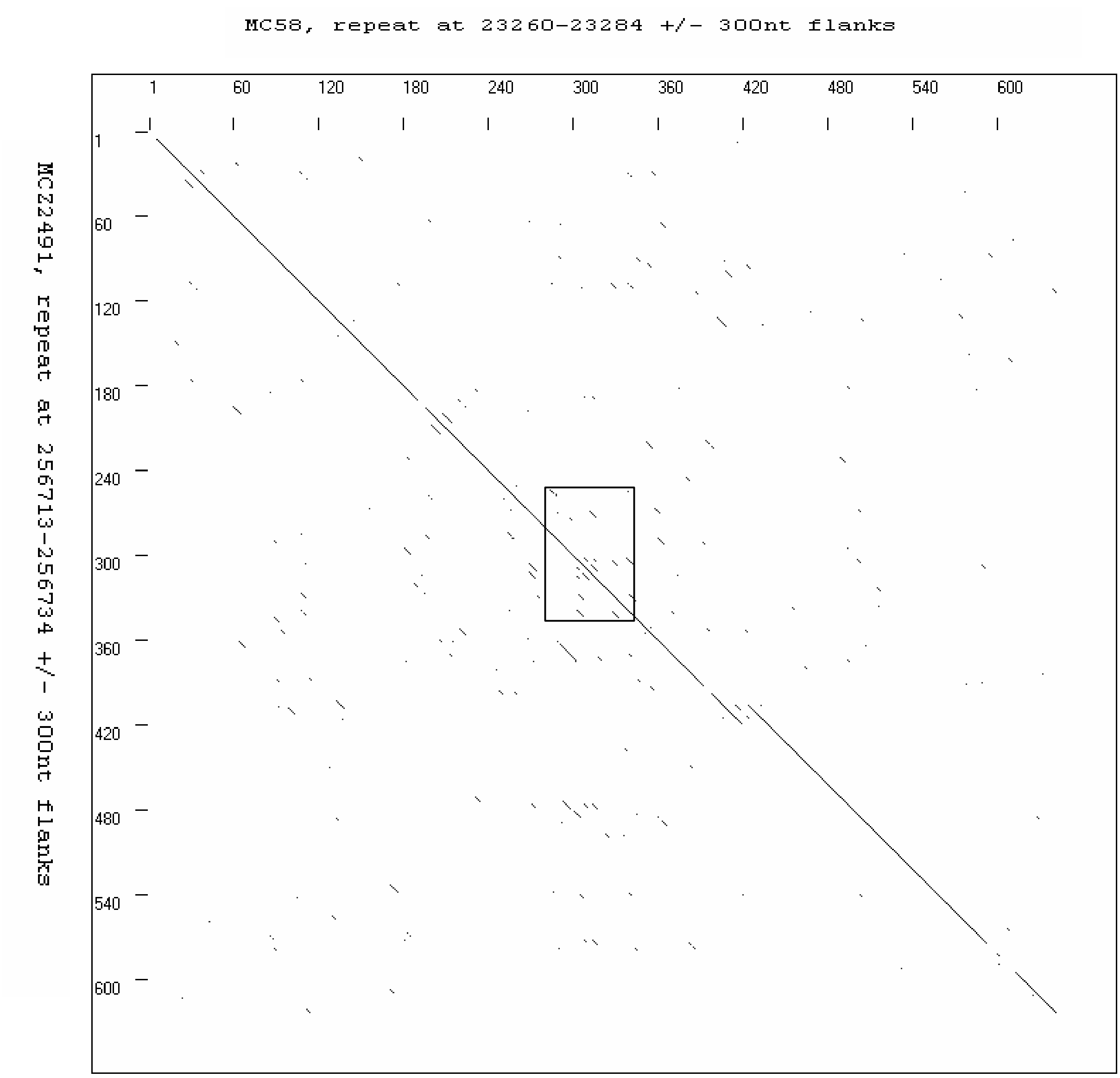

VNTRfinder: 1604964-1605008   1741676-1741720

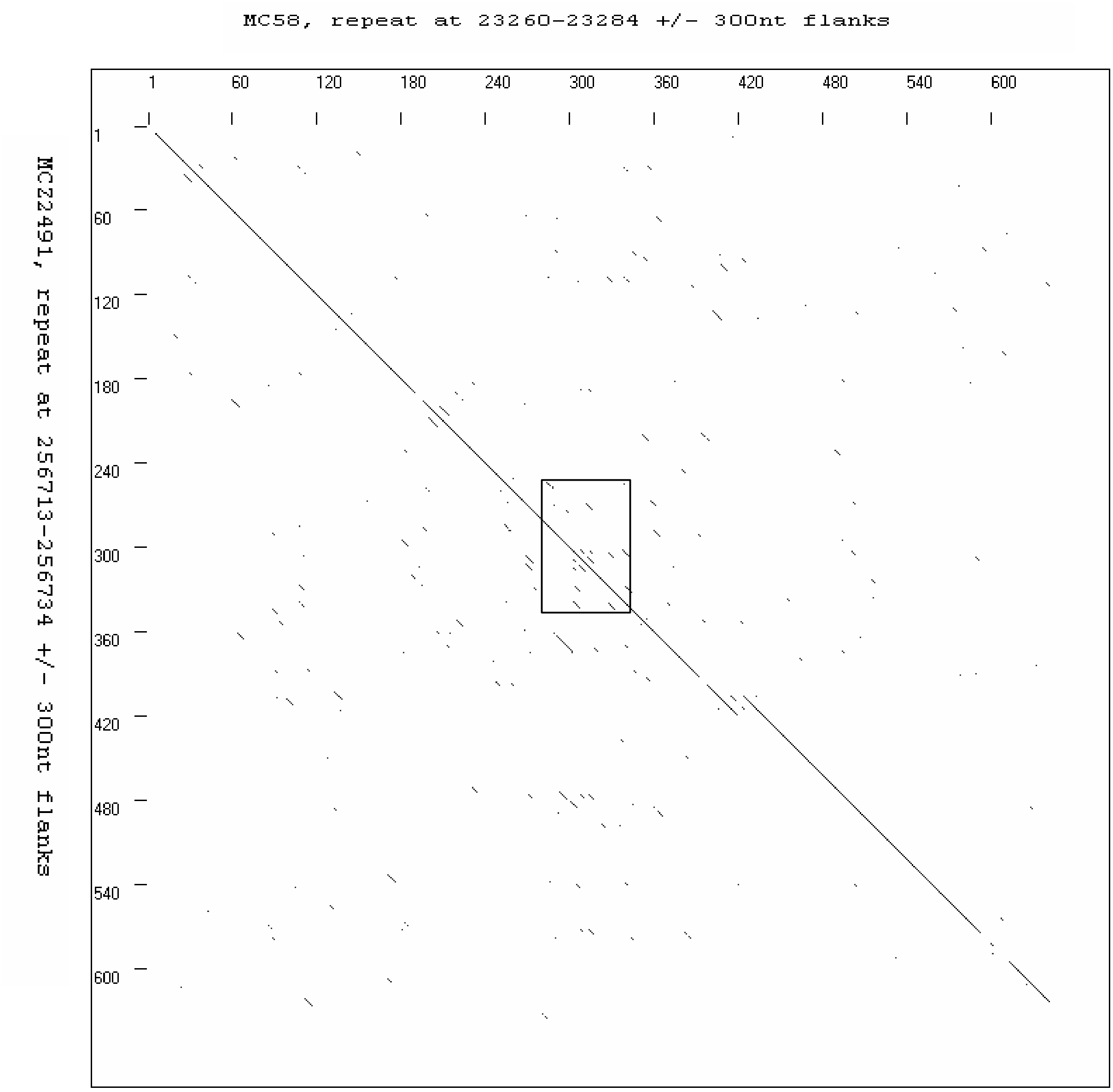

Repeat start-stop: 1694228-1694275
Unit: CGAATCGGGATT
Tandem array:
CGTATCGGGATTTGCATCGGGATTCAAATCGGGGTCGGGTTCGGGATT
Denoeud report variant of 18
VNTRfinder report variant of 0

Denoeud: 1694228-1694275 1741705-1741676

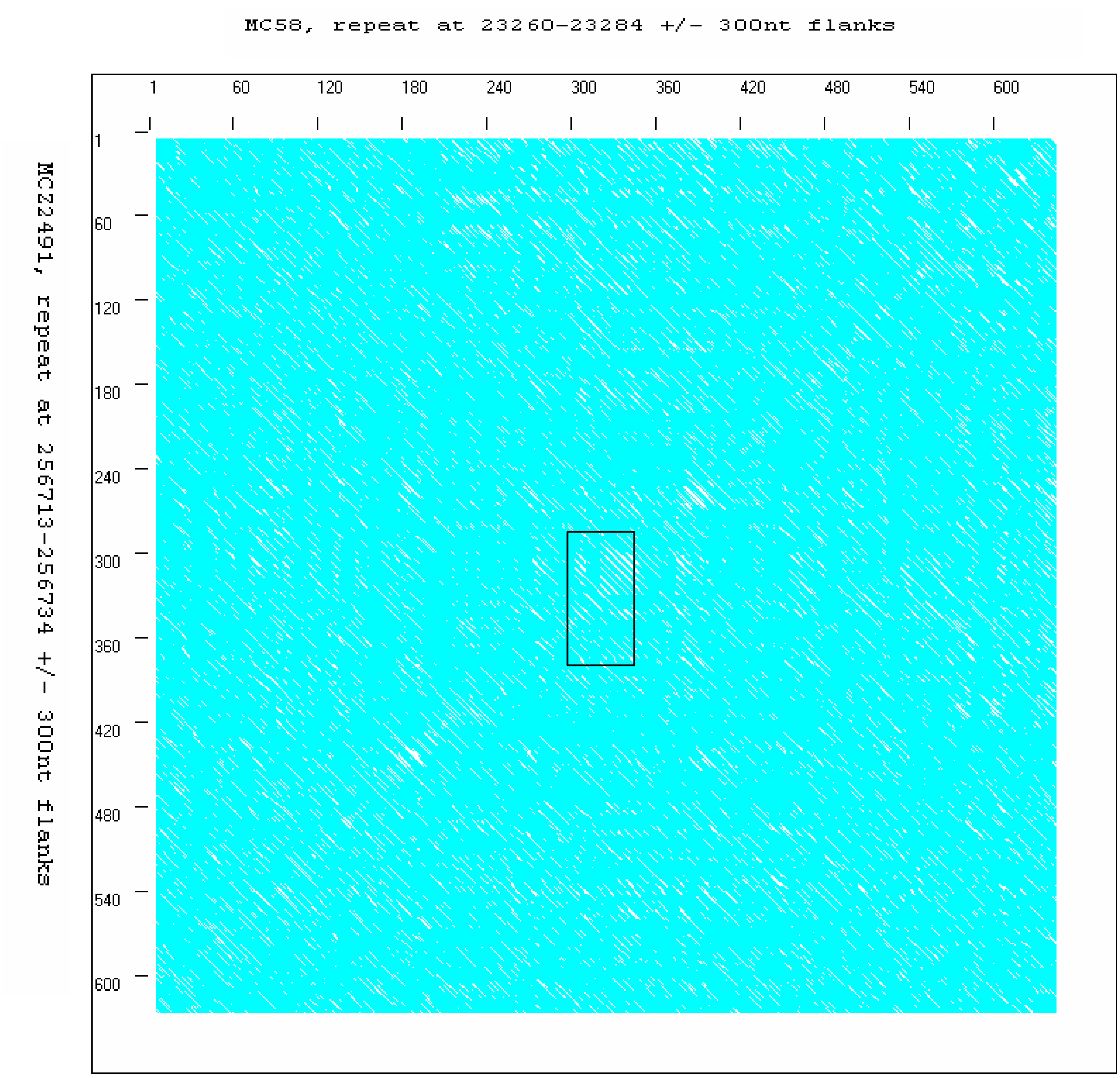

VNTRfinder: 1694228-1694275   1741676-1741723

MC58, repeat at 23260-23284 +/- 300nt flanks

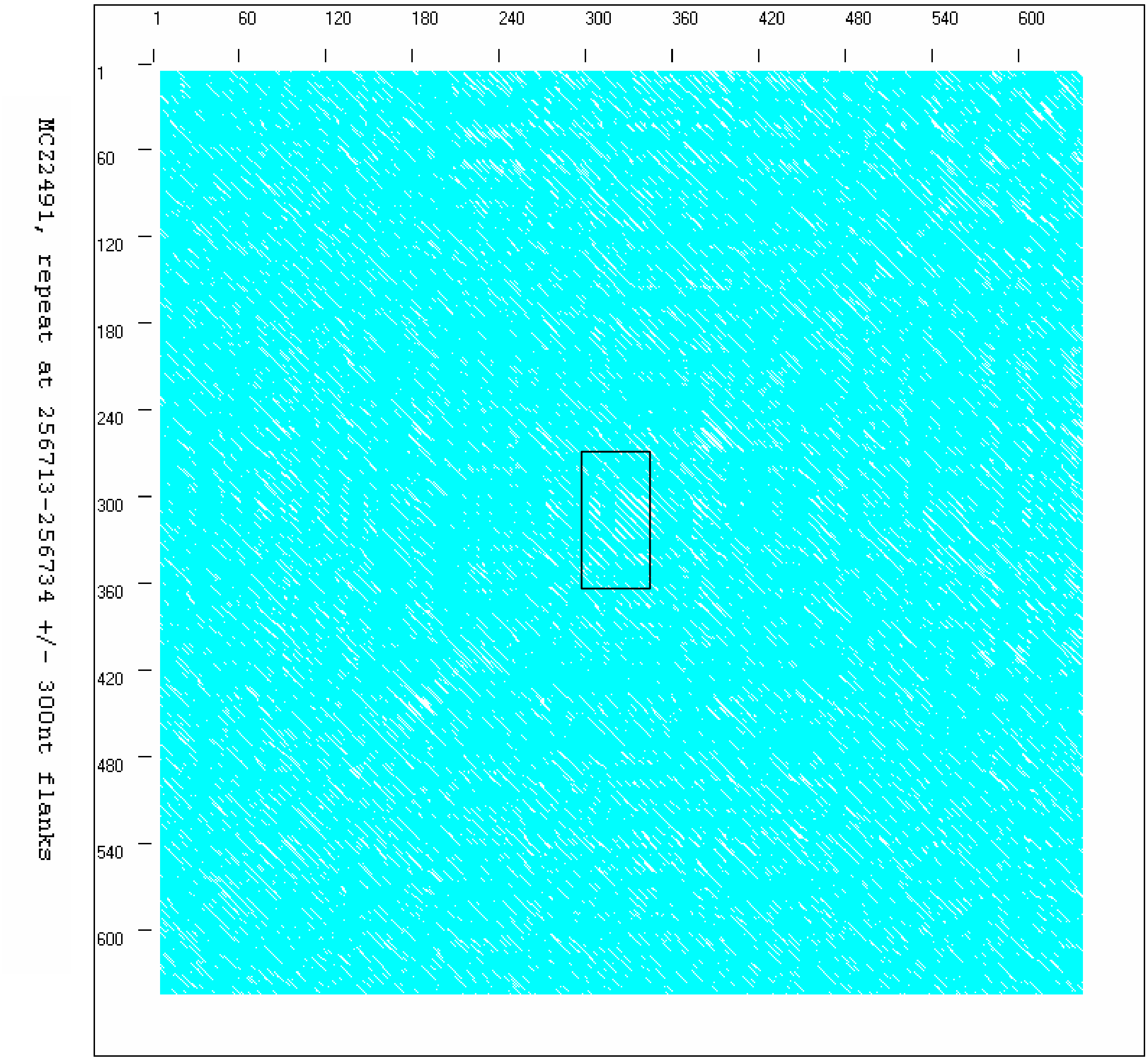

Repeat start-stop: 1822610-1822663
Unit: CCGCCGTCACATTCACTGC
Tandem array:
CCGCCGTGACATTCATGCCCGCCGTCAGATTCACTGCTTTGCCGTCAATATTCA
Denoeud report variant of 134
VNTRfinder report variant of 0

Denoeud: 1822610-1822663 1934569-1934756

MC58, repeat at 23260-23284 +/- 300nt flanks

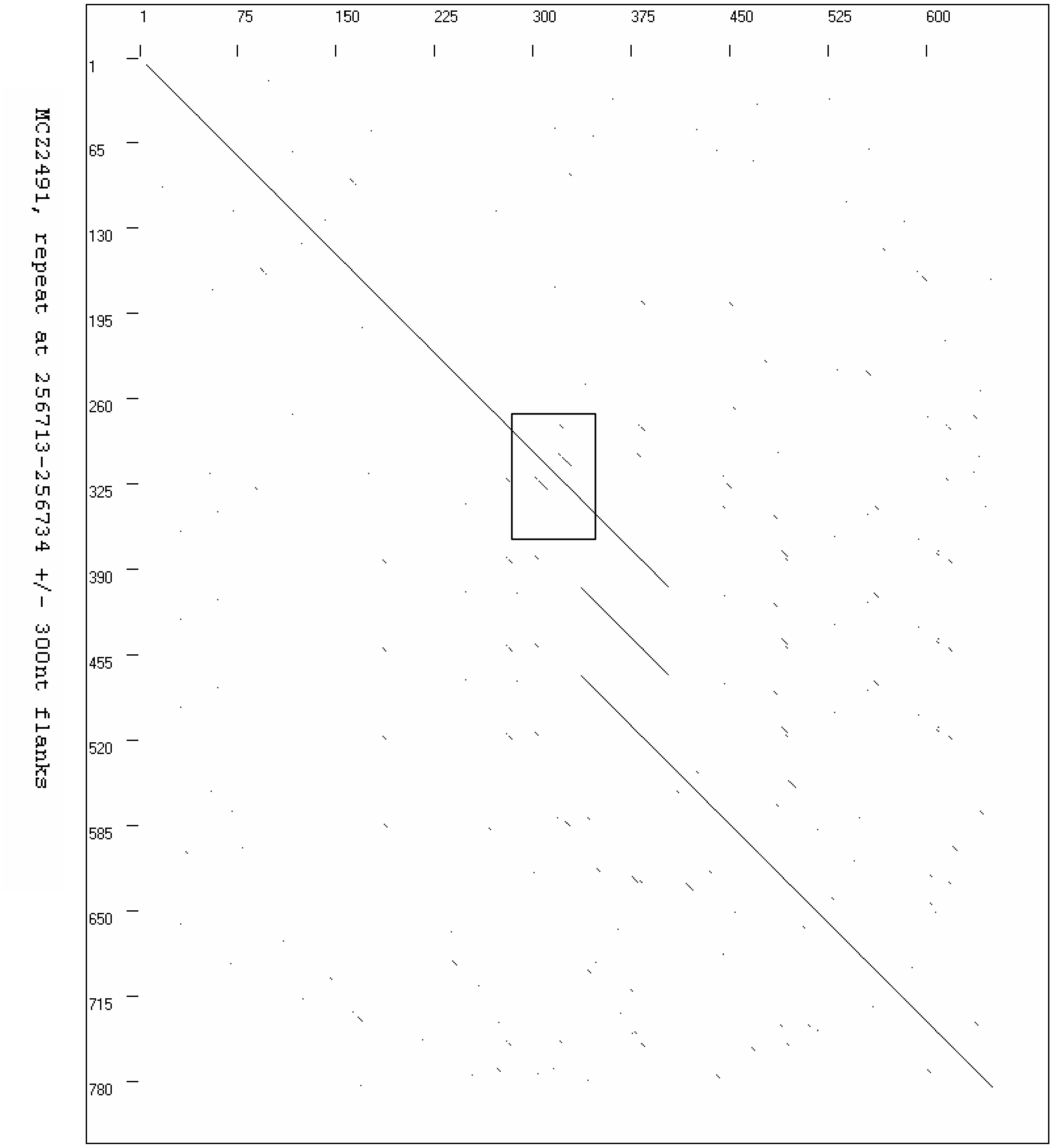

VNTRfinder: 1822610-1822663   1934569-1934622

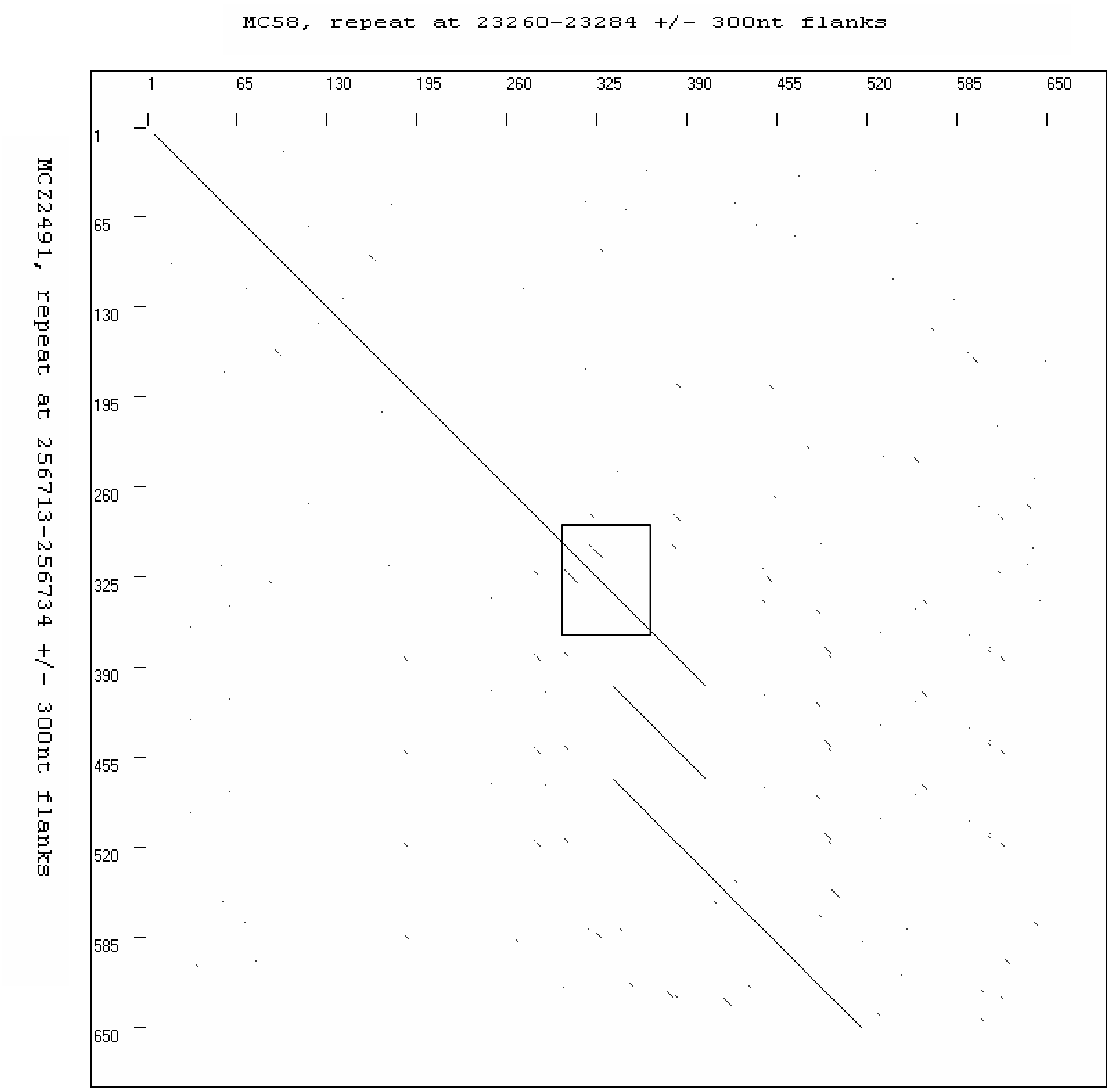

Repeat start-stop: 1975357-1975387
Unit: CTGCCTTCTG
Tandem array: CTGCCTTCTGCTGCCTTTCTCTGACCACTGC
Denoeud report variant of 186
VNTRfinder report variant of 0

Denoeud: 1975357-1975387 558771-558555

MC58, repeat at 23260-23284 +/- 300nt flanks

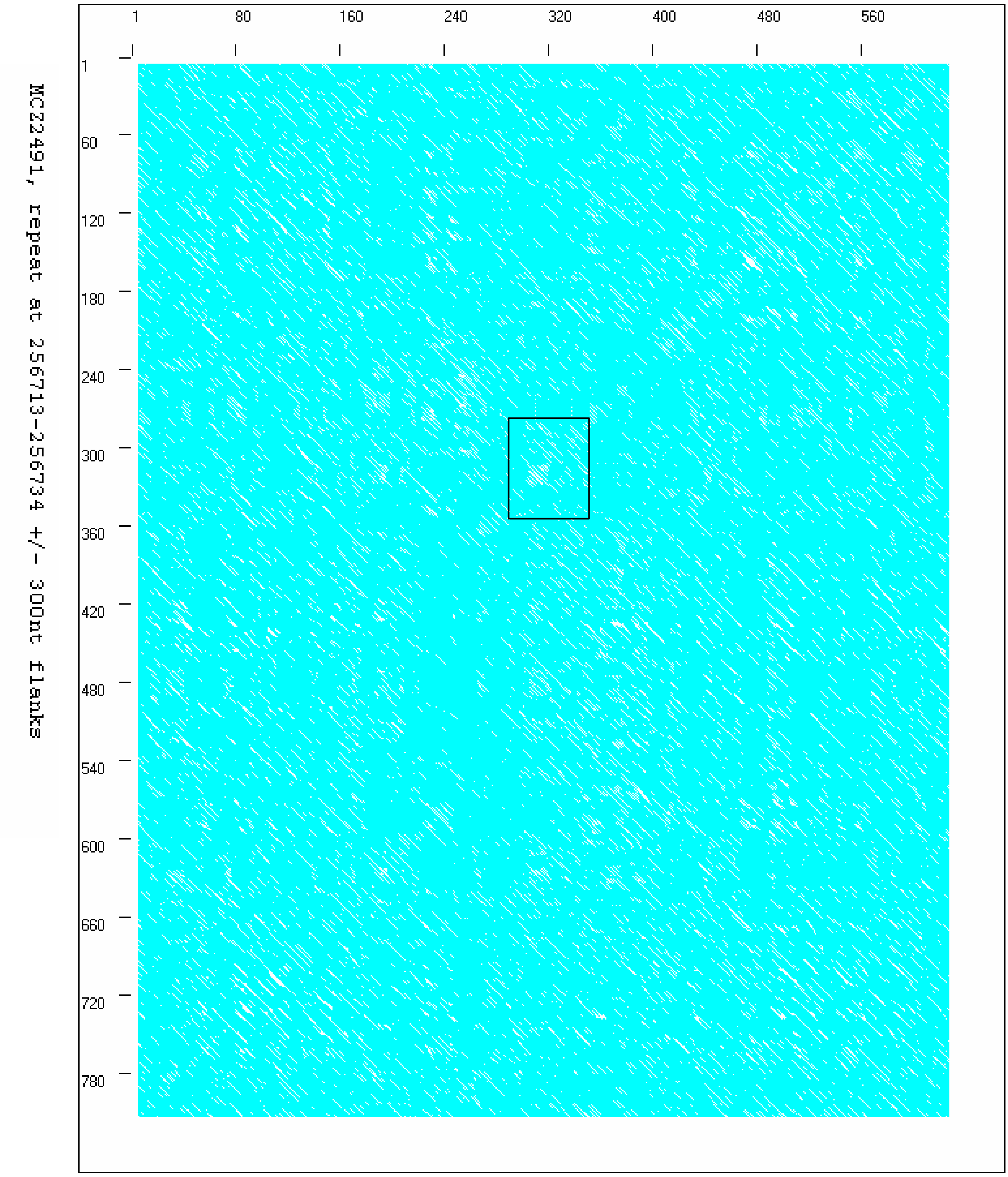

VNTRfinder: 1975357-1975387   558555-558585

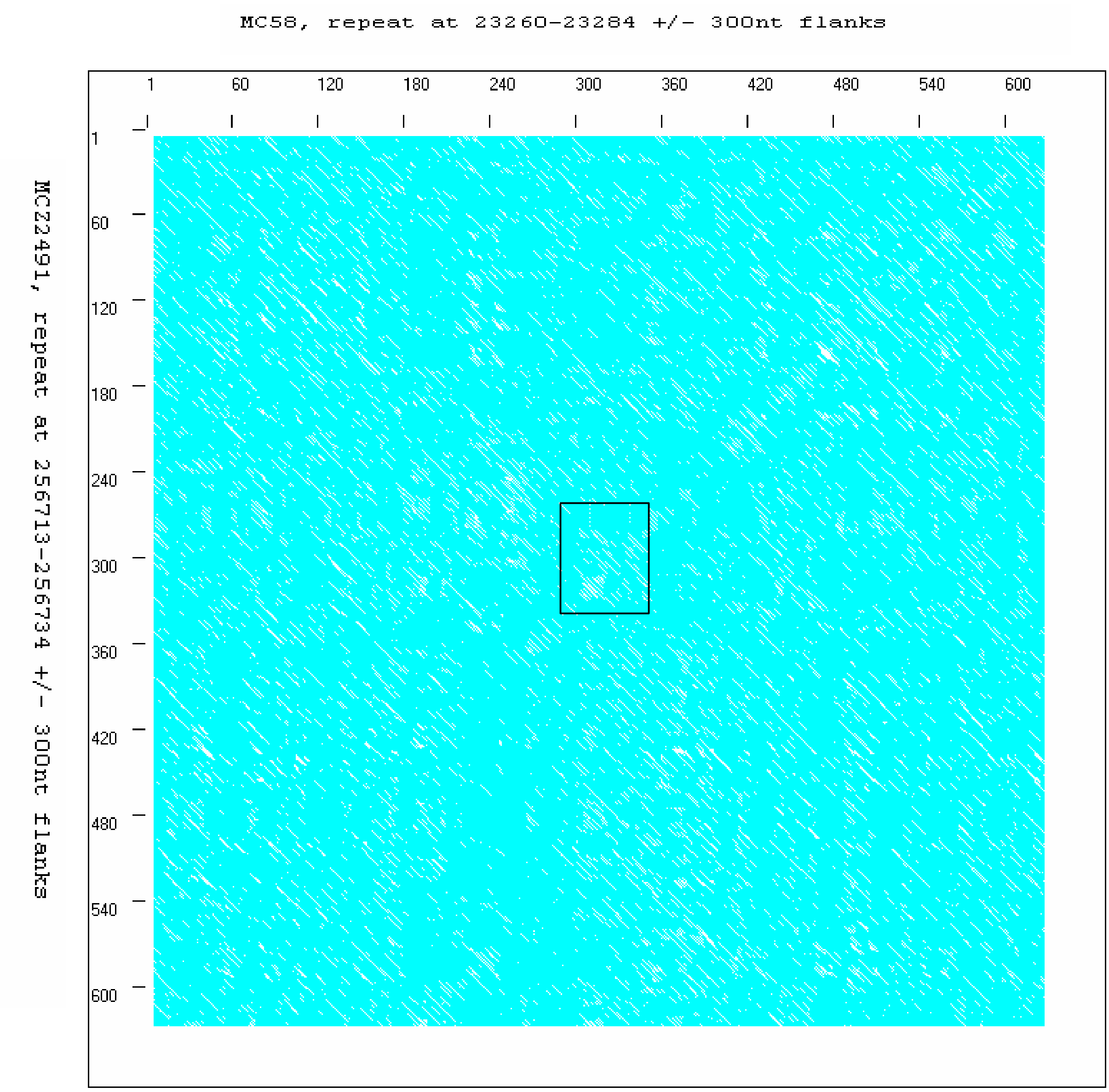

Repeat start-stop: 1985578-1985598
Unit: GGGAGA
Tandem array: GGGAGAGGGTTAGGGAGAGGG
Denoeud report variant of 12
VNTRfinder report variant of 0

Deneoud: 1985578-1985598 547879-547871

MC58, repeat at 23260-23284 +/- 300nt flanks

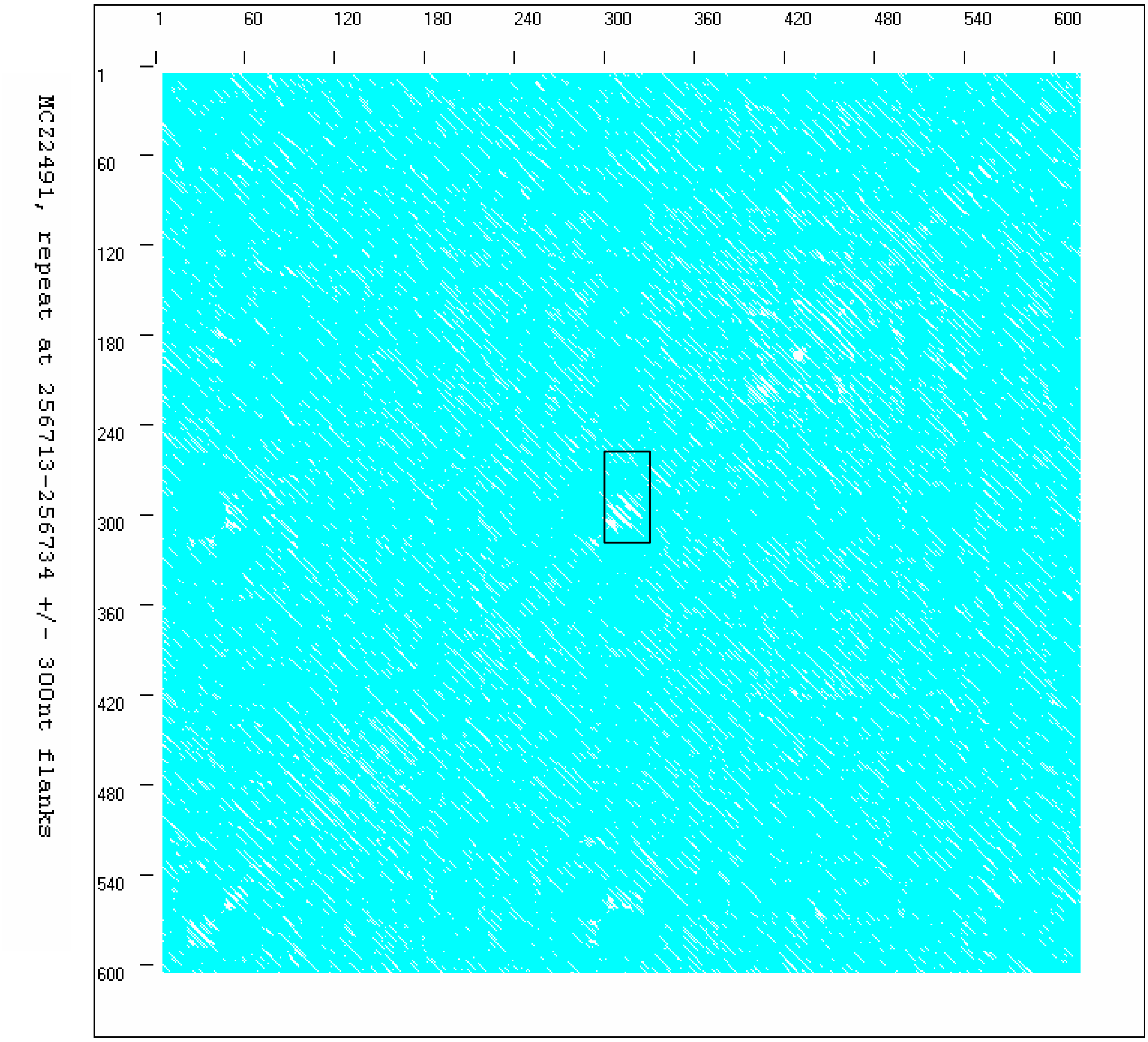

VNTRfinder: 1985578-1985598   547859-547879

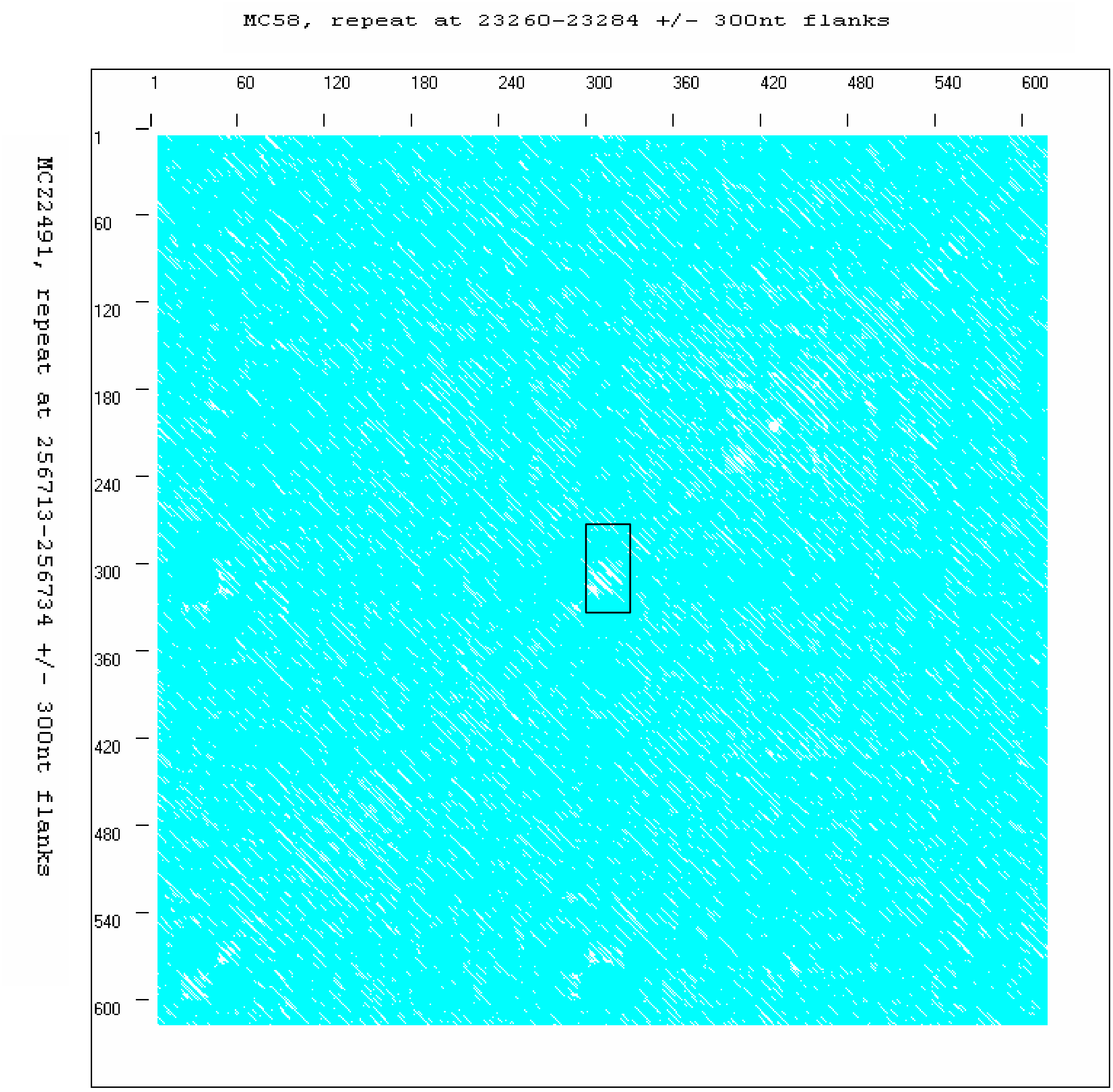
MC58, repeat at 23260-23284 +/- 300nt flanks
MC22491, repeat at 256713-256734 +/- 300nt flanks
1
60
120
180
240
300
360
420
480
540
600
